# An oil containing EPA and DHA from transgenic *Camelina sativa* to replace marine fish oil in feeds for Atlantic salmon (*Salmo salar* L.): Effects on intestinal transcriptome, histology, tissue fatty acid profiles and plasma biochemistry

**DOI:** 10.1371/journal.pone.0175415

**Published:** 2017-04-12

**Authors:** Mónica B. Betancor, Keshuai Li, Matthew Sprague, Tora Bardal, Olga Sayanova, Sarah Usher, Lihua Han, Kjell Måsøval, Ole Torrissen, Johnathan A. Napier, Douglas R. Tocher, Rolf Erik Olsen

**Affiliations:** 1 Institute of Aquaculture, Faculty of Natural Sciences, University of Stirling, Stirling, United Kingdom; 2 Norwegian University of Science and Technology, Department of Biology, Trondheim, Norway; 3 Department of Biological Chemistry and Crop Protection, Rothamsted Research, Harpenden, United Kingdom; 4 Biomar AS, Trondheim, Norway; 5 Institute of Marine Research, Matre, Matredal, Norway; Universidade de Vigo, SPAIN

## Abstract

New *de novo* sources of omega 3 (n-3) long chain polyunsaturated fatty acids (LC-PUFA) are required as alternatives to fish oil in aquafeeds in order to maintain adequate levels of the beneficial fatty acids, eicosapentaenoic and docosahexaenoic (EPA and DHA, respectively). The present study investigated the use of an EPA+DHA oil derived from transgenic *Camelina sativa* in Atlantic salmon (*Salmo salar*) feeds containing low levels of fishmeal (35%) and fish oil (10%), reflecting current commercial formulations, to determine the impacts on tissue fatty acid profile, intestinal transcriptome, and health of farmed salmon. Post-smolt Atlantic salmon were fed for 12-weeks with one of three experimental diets containing either a blend of fish oil/rapeseed oil (FO), wild-type camelina oil (WCO) or transgenic camelina oil (DCO) as added lipid source. The DCO diet did not affect any of the fish performance or health parameters studied. Analyses of the mid and hindgut transcriptomes showed only mild effects on metabolism. Flesh of fish fed the DCO diet accumulated almost double the amount of n-3 LC-PUFA than fish fed the FO or WCO diets, indicating that these oils from transgenic oilseeds offer the opportunity to increase the n-3 LC-PUFA in farmed fish to levels comparable to those found a decade ago.

## Introduction

It is widely recognized that the omega-3 (n-3) long-chain polyunsaturated fatty acids (LC-PUFA), eicosapentaenoic (EPA, 20:5n-3) and docosahexaenoic (DHA; 22:6n-3) acids, have beneficial health effects for humans [1–3]. Consequently, many organisations have published recommended daily intakes of EPA and DHA for human consumers (e.g. [4–5]). Although marine microalgae are the main producers of these fatty acids [6], they are accumulated through the marine trophic chain and, therefore, fish and seafood products are the main sources of n-3 LC-PUFA in human diets. Indeed, with traditional capture fisheries stagnating, aquaculture is the best option to provide these healthy fatty acids as farmed products accounted for up to 73.8 million tons of fish and seafood in 2014 [7] contributing to global n-3 LC-PUFA supply. However, fish meal and fish oil are major raw materials employed in the formulation of aquafeeds, and there is gap between the supply and demand of these marine resources, which are finite and limited on an annual basis [8]. Therefore, this has dictated that they must be increasingly replaced in feeds by alternatives including plant meals and vegetable oils. Therefore, modern, sustainable aquafeeds contain increasing levels of these terrestrial agriculture alternatives that contain no LC-PUFA, which has translated into a substantial reduction in the content of EPA and DHA in the flesh of farmed fish such as salmon over the last decade [9].

Recently, new *de novo* sources of n-3 LC-PUFA has been developed from an oilseed crop, *Camelina sativa*, genetically modified to synthesize either EPA or EPA+ DHA [10]. Two such oils have been evaluated as replacements for fish oil in feeds for Atlantic salmon (*Salmo salar*) [11–13] and gilthead sea bream (*Sparus aurata*) [14], as well as mice [15]. In all the fish feeding trials the use of the new GM-derived oils supported comparable growth performance as well as enhanced deposition of n-3 LC-PUFA in flesh compared to fish fed diets containing wild-type camelina oil. However, fish did not accumulate as high levels of these essential fatty acids as fish fed diets containing 100% fish oil as the single lipid source. The control diets in the previous trials were “gold standard” having high levels of fishmeal and fish oil such that the levels of total n-3 LC-PUFA (including 20:4n-3 and 22:5n-3) were in excess of 20% of total fatty acids, far higher that the EPA+DHA content in current salmon feeds in Norway (around 6% of total fatty acids). Therefore, while the feeds in the previous trials were formulated to test the ability of the new oils to support growth at the same level as in “ideal” high marine feeds, they did not reflect present sustainable feed formulations with high levels of plant proteins and vegetable oils.

In the present trial, the efficacy of the oil containing both EPA and DHA from transgenic *Camelina sativa* was evaluated in Atlantic salmon feeds reflecting current commercial formulations. Thus, feeds were formulated with much lower levels of fishmeal than previously and a 1:1 blend of fish oil and vegetable oil, specifically rapeseed oil, was used in the positive control feed to reflect current practice. The effects of this new *de novo* source of EPA and DHA on fish performance, tissue fatty acid profiles, liver and intestine morphology, liver gene expression as well as mid and hindgut transcriptome were determined. In addition, the effect of an environmental stressor (chasing) on several fish blood and plasma parameters was evaluated.

## Materials and methods

### Production of oil from transgenic Camelina sativa

A construct containing a cassette of seven genes optimized for EPA and DHA synthesis (a Δ6-desaturase from *Ostococcus tauri* (OtΔ6), a Δ6 fatty acid elongase from *Physcomitrella patens* (PSE1) a Δ5-desaturase from *Thraustochytrium* sp. (TcΔ5), a Δ12-desaturase from *Phytophthora sojae* (PsΔ12), an ω3-desaturase from *Phytophthora infestans* (Pi- ω3), a Δ5-elongase from *O*. *tauri* and a Δ4-desaturase from *Emiliania huxleyi*) was used for transformation as described previously [10]. Seeds harvested from transformed plants were illuminated with green LED light and fluorescent seeds identified using a red lens filter. No obvious phenotypic perturbation was observed as a result of modification of the seed oil composition. Full details are provided in [10]. *C*. *sativa* was grown in a containment glasshouse at 23°C day/16°C night. Oil was extracted from seeds by cold-pressing and solvent extraction to maximize yield (PPM, Magdeburg, Germany), and stabilized by the addition of the anti-oxidant ethoxyquin (300 ppm).

### Diets and feeding trial

Three isonitrogenous and isoenergetic diets were formulated to satisfy the nutritional requirements of salmon (Table 1). The diets supplied 45 g kg^-1^ crude protein and 21 g kg^-1^ crude lipid and were manufactured at BioMar Tech-Centre (Brande, Denmark). The diets had the same basal composition with added oil supplied by a 1:1 blend of fish oil/rapeseed oil, wild-type camelina oil or EPA/DHA-camelina oil and named FO, WCO and DCO, respectively. The fatty acid profiles of the oils used in the present trial are presented in S1 Table. A total of 342 post-smolt Atlantic salmon with an average body weight of 122.5 ± 0.3 (mean ± S.D.) were distributed into 9 400 L squared flow-through seawater tanks (38 fish per tank) and fed one of the three experimental feeds (twice daily) in triplicate for 12 weeks. The experimental tanks were equipped with lids fitted with 18W fluorescent light tubes and automatic feeders (Arvo-Tec T drum 2000, www.arvotec.fi), and fish were fed to excess to ensure that feed availability did not restrict growth. All procedures were approved by the Norwegian Animal Experiment Committee (Forsøksdyrutvalget), experiment ID.8089.

**Table 1:** Proximate and fatty acid compositions (percentage of fatty acids) of the three experimental feeds.

	FO	WCO	DCO
*Feed ingredients (%)*			
Fish meal	35.0	35.0	35.0
Soy protein concentrate (60%)	12.4	12.4	12.4
Wheat gluten	5.0	5.0	5.0
Maize gluten	12.0	12.0	12.0
Wheat	14.0	14.0	14.0
Fish oil	10	-	-
Rapeseed oil	10	-	-
Wild-type Camelina oil	-	20	-
EPA+DHA-Camelina oil	-	-	20
Vitamins/Minerals	1.6	1.6	1.6
Amino acids	0.7	0.7	0.7
Yttrium oxide	0.1	0.1	0.1
*Analysed composition*			
Dry matter (%)	93.8	92.2	93.9
Protein (%)	45.2	45.8	44.9
Fat (%)	22.7	19.1	19.4
Ash	7.5	7.5	7.7
*Fatty acid composition (%)*			
Total saturated^1^	12.4	16.6	17.4
Total monoenes^2^	49.4	31.3	19.8
18:2n-6	11.7	12.5	12.8
20:2n-6	0.1	1.7	0.8
20:3n-6	n.d.	n.d.	1.2
20:4n-6	0.2	n.d.	1.8
Total n-6 PUFA^3^	19.7	20.5	25.6
18:3n-3	3.7	30.2	14.0
20:3n-3	n.d.	1.2	0.7
20:4n-3	0.2	n.d.	2.6
20:5n-3	3.1	1.2	6.3
22:5n-3	0.3	n.d.	1.5
22:6n-3	2.8	2.0	6.3
Total n-3 PUFA^4^	11.0	34.9	33.8
Total PUFA	30.8	55.3	59.3
Total n-3 LC-PUFA	6.4	3.2	16.7

^1^Contains 14:0, 16:0, 18:0, 20:0, 22:0 and 24:0;

^2^Contains 16:1n-7, 18:1n-9, 18:1n-7, 20:1n-9, 22:1n-11 and 22:1n-9;

^3^Contains 18:2n-6;

^4^Contains 18:4n-3.

DCO, feed containing EPA+DHA oil from transgenic Camelina; FO, fish oil feed; LC-PUFA, long-chain polyunsaturated fatty acids (sum of 20:4n-3, 20:5n-3 22:5n-3 and 22:6n-3). n.d., not detected; WCO, wild-type Camelina oil feed.

### Stress challenge test and sample collection

After 12 weeks of feeding, blood and tissues were sampled from randomly selected fish following 48 h fasting, either directly (0 h), or 4 h and 24 h after being subjected to an environmental stress (chasing with a stick for 10 min). At each sampling point fish were killed by overdose with metacaine sulphonate (> 150 mg l^-1^, FINQUEL vet., ScanAqua AS, Årnes, Norway) and blood from 4 fish per tank collected via the caudal vein by 1 ml heparinised syringes fitted with 20G needles and whole blood used for haematocrit determination prior to the stress (0 h) and at 4 h and 24 h post challenge. Blood samples were centrifuged at 10,000 g for 3 min to allow blood and plasma to separate and subsequently frozen on dry ice before storing at -80°C until further analysis.

Samples of liver, midgut and hindgut from the same four fish used for blood extraction were collected, stabilised in RNA Later (Sigma, Poole, UK) and stored at -20°C prior to RNA extraction. Additionally samples of liver, mid and hindgut sections of intestine from the same 4 fish were dissected and placed in 4% buffered formaldehyde for histopathological evaluation. The hindgut was dissected from the point where the intestinal diameter increases, the mucosa becomes darker and rings are clearly observed. Samples of muscle (flesh), liver, brain, head kidney, midgut and hindgut from a further 3 fish per tank at 0 h were immediately frozen and stored at– 80°C prior to lipid and fatty acid analyses. All the remaining fish in the tank (31 fish) were killed by overdose with metacaine sulphonate and measured and weighed at the end of the stress challenge (24 h).

### Proximate composition

Feeds were ground before determination of proximate composition according to standard procedures [16]. Moisture contents were obtained after drying in an oven at 110°C for 24 h and ash content determined after incineration at 600°C for 16 h. Crude protein content was measured by determining nitrogen content (N x 6.25) using automated Kjeldahl analysis (Tecator Kjeltec Auto 1030 analyzer, Foss, Warrington, UK) and crude lipid content determined gravimetrically after Soxhlet lipid extraction (Tecator Soxtec system 2050 Auto Extraction apparatus).

### Calculations

Biometric parameters were estimated as follows: Fulton’s condition factor (k) = 100 * (W/L^3^), where W is the final weight (g) and L is the total length (cm). Specific growth rate (SGR) = 100 * (lnWo—ln Wf) * D^-1^, where Wo and Wf are the initial and final weights (tanks means), respectively, and D represents the number of feeding days.

### Plasma analysis

Plasma cortisol analysis was performed by Cortisol Parameter Assay Kits (Cortisol ELISA, RE 52061, IBL, Hamburg, Germany). Plasma ion levels (sodium, potassium, chloride), alkaline phosphatase, glucose and lactate were analysed by MaxMat PL II (MaxMat, Montpellier, France). Osmolality was measured by Fiske^®^ 210 Microsample Osmometer (Advanced Instruments Inc. Norwood, MA, USA).

### Tissue lipid content and fatty acid composition

Samples of flesh, liver, brain, head kidney, midgut and hindgut from three fish per tank were prepared as pooled homogenates (n = 3 per treatment) and total lipid extracted from 1 g by homogenizing in chloroform/methanol (2:1, v/v) using an Ultra-Turrax tissue disrupter (Fisher Scientific, Loughborough, UK), and content determined gravimetrically [17]. Fatty acid methyl esters (FAME) were prepared from total lipid by acid-catalyzed transesterification at 50°C for 16 h [18], and FAME extracted and quantified by a gas chromatograph (AutoSystem XL, Perkin Elmer, Waltham, MA) with Total Chrom Version 6.3.1 software. The system was equipped with an auto-injector (1 μl, inlet temperature 250°C) and a flame ionisation detector (FID, 280°C). The temperature program for the oven was 90°C for 1 min, then raised to 150°C at 30 min-1 and finally raised to 225°C at 3°C min-1 and held for 7 min. Helium was used as the carrier gas and a fused silica capillary column coated with a chemically bonded polyethylene glycol (CP-Wax 52CB, 25 m × 0.25 mm i.d; Varian, Palo Alto, CA) was used. Individual methyl esters were identified by comparison with known standards and a well-characterized fish oil, and also by reference to published data [19].

### RNA extraction and cDNA synthesis

Liver, midgut and hindgut from eighteen individual fish per dietary treatment were homogenized in 1 ml of TriReagent^®^ (Sigma-Aldrich, Dorset, UK), total RNA isolated following manufacturer’s instructions, and quantity and quality determined by spectrophotometry using a Nanodrop ND-1000 (Labtech Int., East Sussex, UK) and electrophoresis using 200 ng of total RNA in a 1% agarose gel. Additionally the Agilent Bioanalyzer with the RNA LabChip kit (Agilent Technologies) was used to analyze approximately 300 ng of total RNA from a randomly selected number of samples (72 samples; 12 samples per treatment and tissue) and provide an RNA integrity number (RIN), which was higher than 8.0 in all samples (average RIN = 8.2). cDNA was synthesized as detailed in [20] and samples pooled to obtain n = 6 per dietary treatment.

### Microarray hybridizations and image analysis

Transcriptome analysis of midgut and hindgut was performed using an Atlantic salmon custom-made oligoarray (ArrayExpress accession number A-MEXP-2065) with 44k features per array on a four-array-per-slide format (Agilent Technologies UK Ltd., Wokingham, UK). A dual-label experimental design using 18 microarrays was employed for the microarray hybridizations with Cy3-labelled test samples competitively hybridized to a common Cy5-labelled pooled-reference per array. The common reference was a pool of equal amounts of amplified RNA from all test samples.

Indirect labelling and hybridization were performed as reported previously [11]. Briefly, 250 ng of total RNA were amplified (TargetAmpTM 1-Round Aminoallyl-aRNA Amplification Kit 101. Epicentre, Madison, Wisconsin, USA) and experimental and pooled reference labelled with Cy3 or Cy5, respectively (GE HealthCare, Little Chalfont, UK). Microarray hybridizations were performed in SureHyb hybridization chambers in a DNA Microarray Hybridization Oven (Agilent Technologies) with 825 ng of Cy3-labelled experimental biological replicate and Cy5-labelled reference pool being combined and total volume made up to 35 μl with nuclease-free water. Scanning was performed at 5 μm resolution using an Axon GenePix 4200AL Scanner (MDS Analytical Technologies, Wokingham, Berkshire, UK). Laser power was kept constant (80%) and PMT adjusted for each channel so that less than 0.1% features were saturated and the mean intensity ratio of the Cy3 and Cy5 signals was close to one. Details of the microarray experiment were submitted to ArrayExpress under accession number E-MTAB-5529.

### Quantitative real time PCR

Expression of candidate genes as well as genes for microarray validation was determined by quantitative PCR (qPCR) in liver, mid and hindgut of fish from all treatments (S2 Table). Results were normalized using reference genes, *hypoxanthine phosphoribosyltransferase 1* (*hprt1*) and *ribosomal protein L2* (*rpl2*), chosen as the most stable according to GeNorm (stability number M = 0.176 and 0.184, respectively). Primers were designed using Primer 3 [21] in regions that included the microarray probes. qPCR was performed using a Biometra TOptical Thermocycler (Analytik Jena, Goettingen, Germany) in 96-well plates in duplicate 20 μl reaction volumes containing 10 μl of Luminaris Color HiGreen qPCR Master Mix (Thermo Scientific), 1 μl of the primer corresponding to the analyzed gene (10 pmol), 3 μl of molecular biology grade water and 5 μl of cDNA, with the exception of the reference genes, which were determined using 2 μl of cDNA. In addition, amplifications were carried out with a systematic negative control (NTC-no template control) containing no cDNA. Standard amplification parameters contained an UDG pre-treatment at 50°C for 2 min, an initial activation step at 95°C for 10 min, followed by 35 cycles: 15 s at 95°C, 30 s at the annealing Tm and 30 s at 72°C.

### Tracking of the DsRed gene in Atlantic salmon anterior and posterior intestine

The absence of transgenic DNA in salmon tissues was confirmed by PCR of DNA extracted from fish midgut and hindgut. Genomic DNA was extracted using REALPURE extraction kit (Valencia, Spain) according to the manufacturer’s instructions. Briefly, tissue samples were incubated in 300 μl of lysis solution overnight at 55°C with 3 μl of Proteinase K. Following incubation, samples were cooled and RNase treatment performed (37°C for 60 min). After protein precipitation, DNA was precipitated by adding 600 μl of isopropanol and hydrated with 5mM Tris. Total DNA was quantified by spectrophotometry and quality determined by electrophoresis as described above. Two primers pairs targeting an endogenous Atlantic salmon gene (growth hormone; *gh*) and a transgene marker for GM–plants (red fluorescent protein, *dsred*) were used (S2 Table). Fifty ng of extracted DNA was used in PCR amplifications that were performed in a final volume of 10 μl, containing 5 μl of MyTaq^™^ HS Mix (Bioline, London, UK). Each set of PCR included a positive control (DNA from genetically modified-Camelina) and a non-template control (NTC).

### Histological evaluation

Transversal sections of liver, midgut and hindgut fixed in 4% buffered neutral-formaldehyde were embedded in paraffin. Four μm sections of the intestines were stained with Alcian Blue/Periodic acid-Schiff (ABPAS) to differ between neutral/mixed and acidic mucosubstances in goblet cells, while liver was stained with PAS. Goblet cells (μm^-2^) were counted by using the Whole Slide Manager and the Count Tool in the stereology program newCAST (Visiopharm, Denmark). The number of neutral/mixed and acidic stained goblet cells were counted at 40x magnification in 10% of a predefined section area within the villi. The 10% fields of interest (FOI) were randomly selected by the program. A semi-quantitative scoring system adapted from [22] was used to independently score six separate parameters of enteritis within the proximal and distal intestines. These parameters are as follows: 1) the abundance of goblet cells (GC) within the villi; 2) the degree of widening of the lamina propria (LP) 3) the abundance eosinophilic granulocytes (EG) within the sub-epithelial mucosa (SEM) and the degree of infiltration into the LP; 4) the thickness of the SEM and 5) the abundance of intra-epithelial lymphocytes (IEL) within the villi. For evaluation of liver morphology, the modified [23] criteria were used, including nuclear, cytoplasmic and intracytoplasmic evaluation. In addition, the presence of carbohydrates in PAS stained sections was evaluated using a four grade examination scheme: 0, not observed; 1, few; 2, medium; 3, severe. A summary of the parameters for the evaluation of both tissues is presented in S3 Table. Sections were scanned with a NanoZoomer SQ (Hamamatsu Photonics Norden, Sweden).

### Statistical analysis

All data are means ± S.D. (n = 3) unless otherwise specified. Percentage data were subjected to arcsin square-root transformation prior to statistical analyses. Data of fish performance, biometry and tissue fatty acid profiles were tested for normality and homogeneity of variances with Levene’s test prior to one-way analysis of variance (ANOVA) followed by a Tukey-Kramer HSD multiple comparisons of means. Data of plasma biochemistry after the challenge test were subjected to a two-way ANOVA test after checking that data were normal and homogeneous. Data from the histological scoring were analysed following the chi-squared analysis for non-parametric data. All statistical analyses were performed using SPSS software (IBM SPSS Statistics 19; SPSS Inc., Chicago, IL, USA).

Statistical analysis of microarray hybridization data was performed in GeneSpring GX version 12.6.1 (Agilent Technologies, Wokingham, Berkshire, UK) using a Welch (unpaired unequal variance) t-test, at 0.05 significance given that often a fraction of the genes show unequal variability between groups [24]. Benjamini-Hochberg multiple test correction was employed. Data were submitted to the Kyoto Encyclopedia of Genes and Genomes (KEGG) [25] for biological function analysis. Gene expression results were analysed using the relative expression software tool (REST 2009; http://www.gene-quantification.info/), which employs a pairwise fixed reallocation randomization test (10,000 randomizations) with efficiency correction to determine the statistical significance of expression ratios (gene expression fold changes) between two treatments [26].

## Results

### Fish growth performance

After 12 weeks of feeding the experimental diets, fish more than doubled their weight, with no differences in fish weight, length or other performance parameters evaluated between fish fed the three dietary treatments (Table 2). No mortality or signs of disease were observed throughout the experimental period.

**Table 2:** Fish performance and survival over the 12-week experimental period.

	FO	WCO	DCO
Initial weight (g)	122.4 ± 2.0	122.4 ± 2.60	122.9 ± 0.7
Initial length (cm)	22.3 ± 0.2	22.4 ± 0.2	22.3 ± 0.1
Final weight (g)	391.4 ± 8.5	406.5 ± 8.6	394.4 ± 14.4
Total length (cm)	31.5 ± 1.4	31.3 ± 1.7	31.7 ± 1.3
Survival (%)	100.0 ± 0.0	100.0 ± 0.0	100.0 ± 0.0
k	1.3 ± 0.0	1.3 ± 0.1	1.2 ± 0.0
SGR (%/day)	1.4 ± 0.0	1.4 ± 0.0	1.5 ± 0.1

DCO, feed containing EPA+DHA oil from transgenic camelina; FO, control (fish oil) feed; k, condition factor; SGR, specific growth rate; WCO, wild-type camelina oil feed. There were no significant differences.

### Lipid contents and fatty acid compositions of tissues

There were no differences in total lipid contents of flesh, brain, head kidney, midgut or hindgut among the dietary treatments (Tables 3–5). In liver, WCO-fed fish displayed the highest lipid contents (p < 0.05), with no difference between fish fed FO or DCO diets (Table 3). The fatty acid compositions showed some tissue-specific differences although they all largely reflected dietary fatty acid compositions (Tables 3–5). In this respect, muscle (flesh) of fish fed diet DCO showed higher proportions of EPA, DPA and DHA as well as n-3 LC-PUFA and n-6 PUFA, than fish fed either FO or WCO (Table 3). This was true also in absolute terms, with DCO-fed fish showing 931 mg of n-3 LC-PUFA per portion (130 g fillet) compared to only 587 mg and 494 mg in fish fed FO or WCO, respectively (S1 Fig). The proportions of EPA were also significantly higher in flesh, head kidney, midgut and hindgut in fish fed DCO than those fed the FO or WCO diets. The percentages of DHA in liver, head kidney, midgut and hindgut were equivalent in fish fed DCO to those fed diet FO. The percentages of DPA were significantly higher in all tissues, other than brain, in fish fed DCO compared to fish fed the other diets. In general, dietary effect on fatty acid composition were not as pronounced in brain as in the other tissues, with no differences among treatments in any of the totals for fatty acid groups or DHA and DPA (Table 4). Differences were observed between midgut and hindgut fatty acid profiles, particularly with DHA, with no differences in levels of this fatty acid between fish fed the three diets in midgut, whereas higher DHA levels were observed in hindgut of fish fed FO and DCO (Table 5).

**Table 3:** Lipid contents (percentage of wet weight) and fatty acid compositions (percentage of total fatty acids) of total lipid of flesh and liver after feeding the experimental diets for 12 weeks.

	FO	WCO	DCO
*Flesh*			
Lipid content	6.3 ± 0.9	5.6 ± 0.4	5.4 ± 0.8
16:0	11.5 ± 0.2^a^	9.6 ± 0.3^c^	10.6 ± 0.2^b^
Total saturated^1^	17.9 ± 0.3^a^	14.9 ± 0.2^c^	17.1 ± 0.2^b^
18:1n-9	25.4 ± 0.1^a^	17.8 ± 0.5^b^	12.5 ± 0.5^c^
Total monoenes^2^	47.7 ± 0.4^a^	33.3 ± 0.6^b^	23.3 ± 0.8^c^
18:2n-6	16.3 ± 0.4^b^	15.3 ± 0.0^c^	18.4 ± 0.1^a^
20:4n-6	0.3 ± 0.0^b^	0.2 ± 0.0^b^	1.5 ± 0.0^a^
Total n-6 PUFA^3^	18.2 ± 0.4^b^	17.9 ± 0.0^b^	23.0 ± 0.1^a^
18:3n-3	2.9 ± 0.1^c^	19.3 ± 0.2^a^	11.0 ± 0.2^b^
20:5n-3	2.2 ± 0.1^b^	1.9 ± 0.0^c^	4.7 ± 0.2^a^
22:5n-3	0.8 ± 0.0^b^	0.6 ± 0.0^c^	2.2 ± 0.0^a^
22:6n-3	6.8 ± 0.8^b^	6.2 ± 0.2^b^	10.4 ± 0.6^a^
Total n-3 PUFA^4^	14.2 ± 0.7^b^	32.6 ± 0.3^a^	33.5 ± 1.0^a^
Total PUFA	32.4 ± 0.3^c^	50.5 ± 0.4^b^	56.5 ± 1.0^a^
EPA + DHA	9.0 ± 0.8^b^	8.1 ± 0.2^b^	15.1 ± 0.7^a^
Total n-3 LC-PUFA	10.4 ± 0.7^b^	9.8 ± 0.3^b^	19.9 ± 0.8^a^
*Liver*			
Lipid content	4.2 ± 0.6^b^	7.1 ± 1.6^a^	4.5 ± 0.5^b^
16:0	15.1 ± 1.7^a^	7.0 ± 0.9^c^	12.7 ± 1.6^b^
Total saturated^1^	21.8 ± 1.5^a^	12.2 ± 1.4^b^	20.8 ± 1.8^a^
18:1n-9	17.7 ± 3.5^a^	22.8 ± 1.0^a^	11.2 ± 1.4^b^
Total monoenes^2^	27.9 ± 6.5^a^	35.0 ± 2.0^a^	17.1 ± 2.3^b^
18:2n-6	9.9 ± 1.7^b^	15.4 ± 1.2^a^	10.5 ± 2.0^b^
20:4n-6	2.7 ± 0.9^b^	0.9 ± 0.1^c^	5.2 ± 0.7^a^
Total n-6 PUFA^3^	16.0 ± 0.3^b^	20.2 ± 1.3^a^	19.7 ± 1.8^a^
18:3n-3	1.4 ± 0.4^c^	14.0 ± 1.0^a^	5.7 ± 0.9^b^
20:5n-3	5.1 ± 1.4^ab^	3.0 ± 0.6^b^	6.6 ± 0.4^a^
22:5n-3	1.7 ± 0.2^b^	0.7 ± 0.2^c^	2.6 ± 0.2^a^
22:6n-3	25.0 ± 4.5^a^	9.1 ± 2.0^b^	22.9 ± 3.5^a^
Total n-3 PUFA^4^	33.9 ± 5.3^ab^	31.5 ± 1.8^b^	41.4 ± 2.6^a^
Total PUFA	50.0 ± 5.2^b^	51.7 ± 0.5^b^	61.1 ± 1.1^a^
EPA + DHA	30.1 ± 5.9^a^	12.1 ± 2.6^b^	29.4 ± 3.9^a^
Total n-3 LC-PUFA	32.3 ± 6.0^a^	14.3 ± 2.9^b^	34.1 ± 3.8^a^

Data expressed as means ± SD (n = 3). Different superscript letters within a row denote significant differences among diets. Statistical differences were determined by one-way ANOVA with Tukey’s comparison test (p < 0.05).

^1^Contains 14:0, 15:0, 18:0, 20:0 and 22:0;

^2^Contains 16:1n-7, 18:1n-7, 20:1n-9, 22:1n-11, 22:1n-9 and 24:1;

^3^Contains 20:2n-6 and 20:3n-6.

^4^Contains 18:4n-3, 20:3n-3 and 20:4n-3.

DCO, feed containing EPA+DHA oil from transgenic Camelina; FO, fish oil feed; LC- PUFA, long-chain polyunsaturated fatty acids (sum of 20:4n-3, 20:5n-3 22:5n-3 and 22:6n-3); WCO, wild-type camelina oil feed.

**Table 4:** Lipid contents (percentage of wet weight) and fatty acid compositions (percentage of total fatty acids) of total lipid of the brain and head kidney (n = 3) after feeding the experimental diets for 12 weeks.

	FO	WCO	DCO
*Brain*			
Lipid content	7.4 ± 0.0	7.2 ± 0.2	7.0 ± 0.6
16:0	16.2 ± 0.4	16.0 ± 0.1	16.1 ± 0.0
Total saturated^1^	24.7 ± 0.9	24.1 ± 0.2	24.4 ± 0.2
18:1n-9	21.0 ± 1.1	20.8 ± 0.4	20.1 ± 0.1
Total monoenes^2^	33.4 ± 1.5	32.3 ± 0.8	31.6 ± 0.3
18:2n-6	2.2 ± 0.4	1.6 ± 0.6	1.7 ± 0.2
20:4n-6	1.1 ± 0.0^b^	1.0 ± 0.0^b^	1.8 ± 0.1^a^
Total n-6 PUFA^3^	3.8 ± 0.4	3.2 ± 0.5	3.9 ± 0.4
18:3n-3	0.4 ± 0.1^b^	1.6 ± 0.6^a^	0.9 ± 0.1^ab^
20:5n-3	5.2 ± 0.0^b^	5.4 ± 0.0^a^	5.4 ± 0.1^a^
22:5n-3	1.9 ± 0.2	2.0 ± 0.0	2.2 ± 0.0
22:6n-3	23.3 ± 0.9	22.6 ± 0.1	23.0 ± 0.4
Total n-3 PUFA^4^	31.0 ± 1.0	32.7 ± 0.8	32.4 ± 0.4
Total PUFA	34.9 ± 1.2	35.9 ± 1.3	36.4 ± 0.1
EPA + DHA	28.5 ± 0.9	28.0 ± 0.1	28.4 ± 0.4
Total n-3 LC-PUFA	30.6 ± 1.0	30.4 ± 0.1	31.1 ± 0.4
*Head kidney*			
Lipid content	3.7 ± 0.5	3.7 ± 0.8	3.3 ± 0.3
16:0	14.2 ± 0.7	13.0 ± 1.6	13.9 ± 1.0
Total saturated^1^	20.9 ± 0.7	19.0 ± 1.7	21.0 ± 1.2
18:1n-9	22.6 ± 1.0^a^	17.4 ± 1.7^b^	14.4 ± 2.0^b^
Total monoenes^2^	40.0 ± 1.5^a^	30.1 ± 3.0^b^	24.6 ± 3.0^b^
18:2n-6	12.9 ± 0.1	12.3 ± 1.1	13.4 ± 0.6
20:4n-6	1.2 ± 0.1^b^	0.9 ± 0.2^b^	3.5 ± 0.6^a^
Total n-6 PUFA^3^	16.0 ± 0.2^b^	15.8 ± 0.7^b^	19.5 ± 0.3^a^
18:3n-3	2.3 ± 0.1^c^	13.0 ± 1.2^a^	6.8 ± 0.3^b^
20:5n-3	3.9 ± 0.5^b^	3.9 ± 0.7^b^	5.4 ± 0.3^a^
22:5n-3	1.0 ± 0.1^b^	0.9 ± 0.1^b^	1.9 ± 0.0^a^
22:6n-3	12.8 ± 1.0	12.0 ± 2.5	14.5 ± 1.3
Total n-3 PUFA^4^	21.2 ± 1.4^b^	33.8 ± 2.0^a^	32.5 ± 1.4^a^
Total PUFA	37.2 ± 1.5^b^	49.6 ± 1.3^a^	52.0 ± 1.6^a^
EPA + DHA	16.7 ± 1.5	15.9 ± 3.2	20.0 ± 1.6
Total n-3 LC-PUFA	18.3 ± 1.6^ab^	17.9 ± 3.4^b^	23.8 ± 1.6^a^

Data expressed as means ± SD (n = 3). Different superscript letters within a row denote significant differences among diets. Statistical differences were determined by one-way ANOVA with Tukey’s comparison test (p < 0.05).

^1^Contains 14:0, 15:0, 18:0, 20:0 and 22:0;

^2^Contains 16:1n-7, 18:1n-7, 20:1n-9, 22:1n-11, 22:1n-9 and 24:1;

^3^Contains 20:2n-6 and 20:3n-6.

^4^Contains 18:4n-3, 20:3n-3 and 20:4n-3.

DCO, feed containing EPA+DHA oil from transgenic Camelina; FO, fish oil feed; LC- PUFA, long-chain polyunsaturated fatty acids (sum of 20:4n-3, 20:5n-3 22:5n-3 and 22:6n-3); WCO, wild-type camelina oil feed.

**Table 5:** Lipid contents (percentage of wet weight) and fatty acid compositions (percentage of total fatty acids) of total lipid of midgut and hindgut (n = 3) after feeding the experimental diets for 12 weeks.

	FO	WCO	DCO
*Midgut*			
Lipid content	5.0 ± 0.7	5.7 ± 1.3	4.4 ± 0.4
16:0	14.7 ± 0.8^a^	12.0 ± 1.4^b^	13.9 ± 0.4^ab^
Total saturated^1^	22.8 ± 1.1^a^	19.2 ± 1.9^b^	23.4 ± 0.9^a^
18:1n-9	19.5 ± 1.3^a^	16.6 ± 1.5^a^	12.0 ± 0.5^b^
Total monoenes^2^	35.3 ± 2.6^a^	29.5 ± 2.9^b^	20.6 ± 1.0^c^
18:2n-6	11.7 ± 1.2	12.5 ± 1.6	12.8 ± 1.0
20:4n-6	1.8 ± 0.2^b^	1.2 ± 0.4^b^	4.0 ± 0.3^a^
Total n-6 PUFA^3^	15.5 ± 1.0^b^	16.1 ± 1.1^b^	19.4 ± 0.9^a^
18:3n-3	2.1 ± 0.2^c^	13.1 ± 2.5^a^	6.5 ± 0.7^b^
20:5n-3	3.4 ± 0.4^b^	3.0 ± 0.5^b^	5.3 ± 0.2^a^
22:5n-3	1.0 ± 0.0^b^	0.9 ± 0.1^c^	1.9 ± 0.0^a^
22:6n-3	18.5 ± 1.7	13.9 ± 4.7	18.7 ± 2.1
Total n-3 PUFA^4^	25.9 ± 1.9^b^	34.5 ± 2.2^a^	35.9 ± 1.1^a^
Total PUFA	41.4 ± 1.0^c^	50.6 ± 1.1^b^	55.4 ± 0.4^a^
EPA + DHA	21.9 ± 2.1	17.0 ± 5.1	24.0 ± 2.1
Total n-3 LC-PUFA	23.2 ± 2.1^ab^	18.7 ± 5.0^b^	27.6 ± 1.9^a^
*Hindgut*			
Lipid content	4.3 ± 0.4	6.1 ± 1.4	4.9 ± 0.4
16:0	13.9 ± 0.6^a^	11.2 ± 0.8^b^	12.9 ± 0.4^a^
Total saturated^1^	22.2 ± 0.7^a^	18.2 ± 1.4^b^	21.2 ± 0.7^a^
18:1n-9	19.2 ± 2.0^a^	17.1 ± 0.9^a^	13.3 ± 1.0^b^
Total monoenes^2^	35.0 ± 3.7^a^	30.6 ± 1.8^a^	22.9 ± 1.4^b^
18:2n-6	10.9 ± 1.4	12.8 ± 1.0	13.2 ± 0.7
20:4n-6	1.3 ± 0.1^b^	0.8 ± 0.2^b^	2.9 ± 0.3^a^
Total n-6 PUFA^3^	14.8 ± 1.2^b^	16.5 ± 0.8^b^	19.4 ± 0.9^a^
18:3n-3	2.0 ± 0.2^c^	13.9 ± 1.5^a^	6.9 ± 0.4^b^
20:5n-3	3.6 ± 0.5^b^	3.1 ± 0.6^b^	5.0 ± 0.3^a^
22:5n-3	1.8 ± 0.3^b^	1.5 ± 0.2^b^	2.6 ± 0.1^a^
22:6n-3	17.9 ± 2.9^a^	11.4 ± 2.4^b^	16.2 ± 0.7^ab^
Total n-3 PUFA^4^	26.3 ± 3.4^b^	34.2 ± 1.5^a^	34.9 ± 1.1^a^
Total PUFA	41.1 ± 2.5^b^	50.6 ± 0.9^a^	54.2 ± 1.7^a^
EPA + DHA	21.5 ± 3.3^a^	14.5 ± 3.0^b^	21.2 ± 0.9^a^
Total n-3 LC-PUFA	23.7 ± 3.5^ab^	17.0 ± 3.2^b^	25.7 ± 1.0^a^

Data expressed as means ± SD (n = 3). Different superscript letters within a row denote significant differences among diets. Statistical differences were determined by one-way ANOVA with Tukey’s comparison test (p < 0.05).

^1^Contains 14:0, 15:0, 18:0, 20:0 and 22:0;

^2^Contains 16:1n-7, 18:1n-7, 20:1n-9, 22:1n-11, 22:1n-9 and 24:1;

^3^Contains 20:2n-6 and 20:3n-6.

^4^Contains 18:4n-3, 20:3n-3 and 20:4n-3.

DCO, feed containing EPA+DHA oil from transgenic Camelina; FO, fish oil feed; LC- PUFA, long-chain polyunsaturated fatty acids (sum of 20:4n-3, 20:5n-3 22:5n-3 and 22:6n-3); WCO, wild-type camelina oil feed

### Plasma biochemistry

Two-way ANOVA showed that the factor “time” was significant for all the parameters, whereas significant differences with “diet” were only observed for chloride and alkaline phosphatase (ALP) (Table 6). Additionally an interaction between “diet” and “time” was observed for osmolarity (p = 0.024).

**Table 6:** Two-way ANOVA results of the plasma parameters in fish at the end of the feeding trial and after the challenge test.

	0 h			1 h			24 h			Significance (p)		
	FO	WCO	DCO	FO	WCO	DCO	FO	WCO	DCO	Diet	Time	DxT
Haematocrit	34.7 ± 5.1	33.6 ± 3.7	30.0 ± 6.8	35.0 ± 5.5	37.1 ± 2.2	36.5 ± 3.7	32.1 ± 4.5	33.5 ± 2.6	31.4 ± 5.1	n.s.	**	n.s.
Cortisol (ng/ml)	80.5 ± 42.3	63.1 ± 31.6	81.1 ± 43.0	587.0 ± 138.9	528.1 ± 170.1	647.2 ± 193.3	77.1 ± 47.7	95.3 ± 38.0	113.6 ± 33.2	n.s.	**	n.s.
Sodium (mmol/l)	158.1 ± 3.8	157.2 ± 3.8	157.9 ± 3.3	196.2 ± 6.0	194.8 ± 5.2	193.7 ± 6.7	164.6 ± 3.4	164.4 ± 4.8	162.5 ± 3.0	n.s.	**	n.s.
Potassium (mmol/l)	4.2 ± 0.5	4.0 ± 0.4	4.1 ± 0.5	4.9 ± 0.6	4.8 ± 0.5	5.2 ± 0.4	3.6 ± 0.3	3.7 ± 0.4	3.7 ± 0.3	n.s.	**	n.s.
Chloride (mmol/l)	134.1 ± 3.6	133.6 ± 2.6	132.7 ± 3.0	167.8 ± 6.0	166.6 ± 4.6	163.8 ± 6.7	141.0 ± 3.5	142.1 ± 4.6	138.7 ± 3.1	*	**	n.s.
mOsm	336.1 ± 6.7	335.2 ± 5.6	349.7 ± 18.1	419.1 ± 13.5	413.2 ± 14.6	416.7 ± 17.3	344.5 ± 8.0	346.3 ± 8.6	341.1 ± 3.3	n.s.	**	*
Alkaline phosphatase (IU)	396.7 ± 97.3	467.7 ± 90.7	522.5 ± 157.4	397.2 ± 73.4	439.6 ± 129.2	439.6 ± 129.2	208.7 ± 77.0	249.9 ± 100.8	208.7 ± 77.0	*	**	n.s.
Lactate (mg/dl)	13.8 ± 3.3	18.0 ± 4.9	15.5 ± 4.7	86.0 ± 28.0	79.4 ± 16.8	79.4 ± 16.8	5.6 ± 2.4	6.3 ± 2.7	185.0 ± 50.0	n.s.	**	n.s.
Glucose (mmol/l)	3.5 ± 0.3	3.8 ± 0.4	3.6 ± 0.6	6.0 ± 0.9	5.6 ± 0.8	5.6 ± 0.8	3.7 ± 0.4	3.9 ± 0.430	249.9 ± 100.8	n.s.	**	n.s.

D, Diet; T, Time.

* p<0.05;

** p<0.01;

n.s. not significant.

### Histological evaluation

There were no significant differences in the number of goblet cells (GC) among the dietary treatments in either mid (p = 0.451) or hindgut (p = 0.369) (Table 7). No significant differences were observed between treatment groups in GC colour in mid or hindguts. There appeared to be a higher number of magenta GC present in midgut in DCO-fed fish, whereas the number of these cells seemed to be higher in hindgut in fish fed WCO, albeit these trends were not significant. No differences were observed in any of the other assessed parameters in either mid or hindgut. Liver of salmon fed WCO showed lower scores in terms of hepatocyte cytoplasm (LHC; p = 0.018) and hepatic lipid intracytoplasmic vacuolization (HV; p = 0.017), with no differences in liver nuclei (LN; p = 0.076; Table 7). Liver PAS staining also differed among fish fed the dietary treatments with fish fed FO showing the highest score (p = 0.031).

**Table 7:** Individual score and overall mean for the different parameter used to assess the midgut, hindgut and the liver in Atlantic salmon fed the three experimental feeds.

		FO	WCO	DCO
*Midgut*	MF	1.2 ± 0.3	1.7 ± 1.2	1.7 ± 0.6
	GC	1.3 ± 0.6	2.0 ± 1.0	2.2 ± 0.8
	LP	1.0 ± 0.0	1.2 ± 0.3	1.0 ± 0.0
	SNV	1.0 ± 0.0	1.3 ± 0.6	1.0 ± 0.0
	EG	1.3 ± 0.3	1.0 ± 0.0	1.2 ± 0.3
	SM	1.2 ± 0.3	1.2 ± 0.3	1.2 ± 0.3
	IEL	1.2 ± 0.3	1.0 ± 0.0	1.2 ± 0.3
	Mean score	9.2 ± 0.3	10.3 ± 0.6	10.3 ± 1.4
*Hindgut*	MF	1.0 ± 0.0	1.0 ± 0.0	1.0 ± 0.0
	GC	1.0 ± 0.0	0.3 ± 0.6	1.2 ± 0.3
	LP	1.0 ± 0.0	1.0 ± 0.0	1.1 ± 0.0
	SNV	1.0 ± 0.0	1.8 ± 1.0	1.2 ± 0.3
	EG	1.3 ± 0.3	1.0 ± 0.3	1.0 ± 0.3
	SM	1.0 ± 0.0	1.0 ± 0.0	1.0 ± 0.0
	IEL	1.2 ± 0.3	1.5 ± 0.5	1.0 ± 0.0
	Mean score	8.5 ± 0.5	9.7 ± 2.1	8.8 ± 0.6
*Liver*	LN	1.0 ± 0.0	1.7 ± 0.6	1.0 ± 0.0
	LHC	1.0 ± 0.0^b^	1.8 ± 0.3^a^	1.2 ± 0.3^b^
	HV	1.0 ± 0.0^b^	2.5 ± 0.5^a^	1.2 ± 0.3^b^
	Glycogen	2.7 ± 0.6^a^	1.2 ± 0.3^b^	0.2 ± 0.3^b^

DCO, feed containing EPA+DHA oil from transgenic Camelina; EG, eosinophilic granulocytes; FO, fish oil feed; GC, goblet cells; HV, hepatic vacuolation; IEL, intraepithelial lymphocytes; LHC, liver hepatocyte cytoplasm; LN, liver nuclei; LP, lamina propria; MF, mucosal folds; SM, sub-epithelial mucosa; SNV, supranuclear vacuoles; WCO, wild-type camelina oil feed.

### Midgut transcriptome

**Fig 1:**
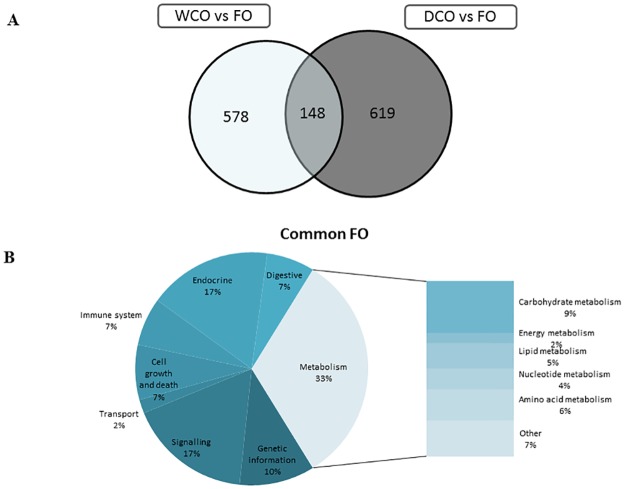
Impact of diet on midgut transcriptome of Atlantic salmon fed camelina oils (DCO and WCO) in comparison with fish fed Fish Oil (FO). **(A) Venn diagram representing mRNA transcripts differentially expressed in the midgut of Atlantic salmon fed the experimental diets DCO and WCO compared to diet FO.** The area of the circles is scaled to the number of transcripts (Welch t-test; p < 0.05; FC > 1.3). (B) Distribution by categories of common differentially genes in midgut between Atlantic salmon fed DCO and WCO when compared to FO-fed fish (Welch t-test; p < 0.05; FC > 1.3). Non-annotated genes and features corresponding to the same gene are not represented.

In midgut, a total of 1319 genes were differentially expressed in salmon fed FO versus DCO whereas 1135 where affected when comparing FO-fed fish with WCO-fed fish. A smaller number (924) of differentially expressed genes (DEG) was found when comparing the midgut of salmon fed DCO against WCO-fed fish. No obvious differences were observed regarding the intensity (fold change; FC) or direction of change (up or down-regulation) between the different contrasts (Table 8). Restricting analysis to probes with a FC over 1.3 revealed 726 and 767 DEG in fish fed FO compared to fish fed WCO and DCO, respectively, with 148 DEG common to both contrasts (Fig 1A). Assigning KEGG Orthology (KO) numbers to these 148 common DEG and mapping them to a known compendium of metabolic pathways (KEGG) indicated that over 59% were annotated, and showed that the metabolism category was highly affected (33%) and, within metabolism, the main subcategories affected were carbohydrate (9%), amino acids (6%) and lipid (5%) (Fig 1B).

**Table 8:** Summary of the results of midgut and hindgut microarray analysis.

	FO/WCO	FO/DCO	DCO/WCO
*Midgut*			
Total no. of probes	44000		
Total no. of DEG	1319	924	1135
**Up-regulated genes**	**584 (44.3%)**	**412 (44.6%)**	**576 (50.7%)**
FC > 1.3	372 (63.7%)	332 (80.6%)	254 (44.1%)
**Down-regulated genes**	**735 (55.7%)**	**512 (55.4%)**	**559 (49.3%)**
FC > 1.3	354 (48.2%)	435 (85.0%)	319 (57.1%)
*Hindgut*			
Total no. of probes	44000		
Total no. of DEG	999	1289	648
**Up-regulated genes**	**650 (65.1%)**	**625 (48.5%)**	**366 (56.4%)**
FC > 1.3	563 (86.6%)	405 (64.8%)	304 (83.1%)
**Down-regulated genes**	**349 (34.9%)**	**664 (51.5%)**	**282 (43.6%)**
FC > 1.3	268 (76.8%)	462 (69.6%)	231 (81.9%)

DCO, feed containing oil from transgenic camelina; DEG, differentially expressed genes; FC, fold change; FO, control (fish oil) feed; WCO, feed containing oil from wild-type camelina.

**Fig 2:**
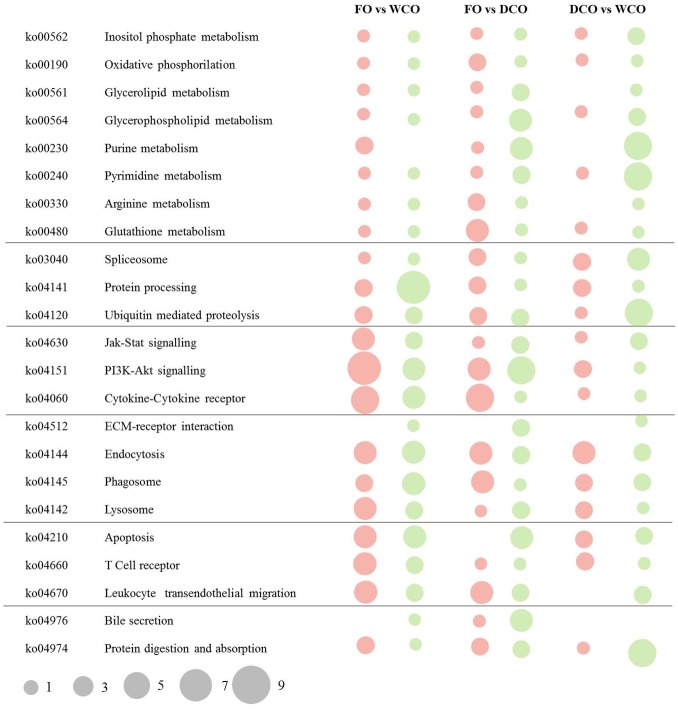
Ranking of differentially expressed pathways in midgut of Atlantic salmon between fish fed a feed based either on Fish Oil (FO), Wild type Camelina Oil (WCO) or EPA+DHA Camelina Oil (DCO). **Pathway analysis was performed using the Kyoto Encyclopedia of Genes and Genome (KEGG).** The area of the circle is scaled to the number of DEG within each pathway. Up-regulated genes, red; down-regulated genes, green.

The pathways with the highest numbers of DEGs in the three contrasts (FO vs. WCO, FO vs. DCO and DCO vs. WCO) were compared (Fig 2). Although the metabolism category was highly represented, a limited number (1–9) of genes was differentially expressed in each pathway, being most numerous in pathways related to protein metabolism such as purine and pyrimidine metabolism. In both these pathways there was strong down-regulation in the midgut of fish fed diet WCO compared to DCO-fed fish. Within the signalling category, the PI3K-Akt signalling and cytokine-cytokine receptor interaction pathways displayed a high of number of DEG were observed between fish fed diets containing WCO or DCO and those fed FO. Indeed, 11 genes belonging to cytokine-cytokine receptor interactions were regulated in the contrast FO vs. WCO, whereas only 2 and 4 were regulated in fish fed DCO compared to FO and WCO, respectively. Most of the DEG belonged to the type I receptors family (hematopoietins), chemokines receptors family (subfamilies CXC and CC), as well as tumour necrosis factor and transforming growth factor beta. Pathways that showed differences between the three contrasts were ubiquitin mediated proteolysis and protein digestion and absorption, which showed high numbers of down-regulated DEG when DCO-fed fish were compared to WCO-fed fish. Within the protein digestion and absorption pathway, two membrane transport proteins, solute carrier family 6 and 15, were down-regulated in DCO-fed fish compared to fish fed WCO (-1.4 and -2.3, respectively).

**Fig 3:**
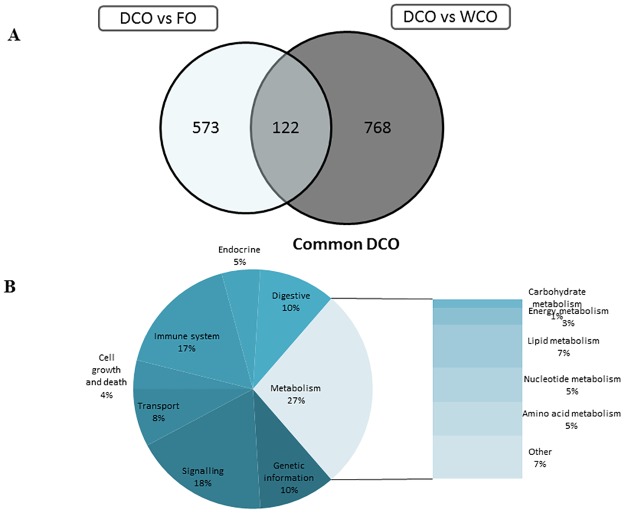
Impact of diet on midgut transcriptome of Atlantic salmon fed diets containing Fish Oil (FO) or Wild type Camelina Oil (WCO) in comparison with fish fed the EPA+DHA Camelina Oil (DCO). (A) Venn diagram representing transcripts differentially expressed in the midgut of Atlantic salmon fed diets FO and WCO compared to diet DCO. The area of the circles is scaled to the number of transcripts (Welch t-test; p < 0.05; FC > 1.3). (B) Distribution by categories of common differentially genes in midgut between Atlantic salmon fed FO and WCO compared to DCO-fed fish (Welch t-test; p < 0.05; FC > 1.3). Non-annotated genes and features corresponding to the same gene are not represented.

To elucidate which genes in midgut were specific to DCO, 122 DEG common to both DCO vs. FO and DCO vs. WCO contrasts were identified at a FC > 1.3 (p < 0.005; Fig 3A). After removing non-annotated genes and duplicated genes, KEGG analysis showed that metabolism was the main category affected (27%) followed by signalling and immune system (18% and 17%, respectively; Fig 3B). Within metabolism, lipid metabolism was the main subcategory affected (7%). All the annotated features within the 122 commonly affected DEG are presented in S4 Table.

### Hindgut transcriptome

**Fig 4:**
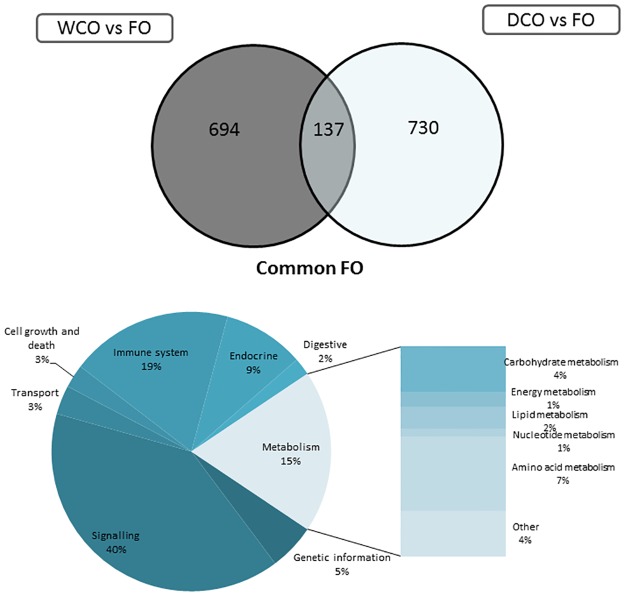
Impact of diet on hindgut transcriptome of Atlantic salmon fed diets containing camelina oils (WCO and DCO) in comparison with fish fed Fish Oil (FO). **(A) Venn diagram representing mRNA transcripts differentially expressed in the hindgut of Atlantic salmon fed diets WCO and DCO compared to diet FO.** The area of the circles is scaled to the number of transcripts (Welch t-test; p < 0.05; FC > 1.3). (B) Distribution by categories of common differentially genes in hindgut between Atlantic salmon fed WCO and DCO when compared to FO-fed fish (Welch t-test; p < 0.05; FC > 1.3). Non-annotated genes and features corresponding to the same gene are not represented.

A similar pattern of DEG between the dietary treatments was observed in hindgut, albeit the number of DEG was lower than in midgut at 999 (FO vs. WCO), 1289 (FO vs. DCO) and 648 (DCO vs. WCO) (Table 8). More DEG were up-regulated than down-regulated when the hindgut of WCO-fed fish was compared with that of fish fed FO and DCO. Restricting DEG to those with a FC > 1.3, 831 probes were differentially expressed in the FO vs. WCO contrast, and 867 in the FO vs. DCO contrast (Fig 4A). KEGG analysis showed that 65.0% of the DEG were annotated. When hindgut of FO-fed fish was compared with that of fish fed either WCO or DCO, signalling was the main category affected (40%; Fig 4B), followed by immune system (19%). Metabolism (15%) was the next most represented category, with amino acid metabolism being the main subcategory affected with lipid metabolism accounting for only 2% of the common DEG (7%; Fig 4B).

**Fig 5:**
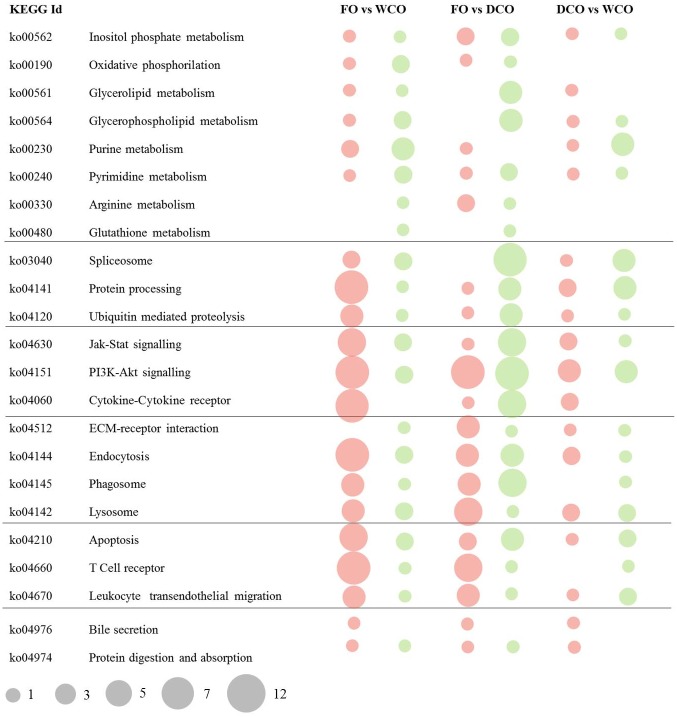
Ranking of differentially expressed pathways in midgut of Atlantic salmon between fish fed a diet containing Fish Oil (FO), Wild type Camelina Oil (WCO) or EPA+DHA Camelina Oil (DCO). Pathway analysis was performed using the Kyoto Encyclopedia of Genes and Genome (KEGG). The area of the circle is scaled to the number of DEG within each pathway. Up-regulated genes, red; down-regulated genes, green.

The same pathways highly represented in the three contrasts in midgut were also observed to have a high number of DEG in hindgut (Fig 5). Within metabolism, a strong down-regulation (5 DEG) in glycerolipid and glycerophospholipid metabolism pathways was observed in the contrast FO vs. DCO. Strong up-regulation (up to 15 genes) in pathways related to signalling, cellular processes and immune system was observed in the FO vs. WCO contrast. A clear trend in these pathways was not found in the other two contrasts. The lowest number of DEG was observed when comparing the hindgut of fish fed WCO and DCO.

**Fig 6:**
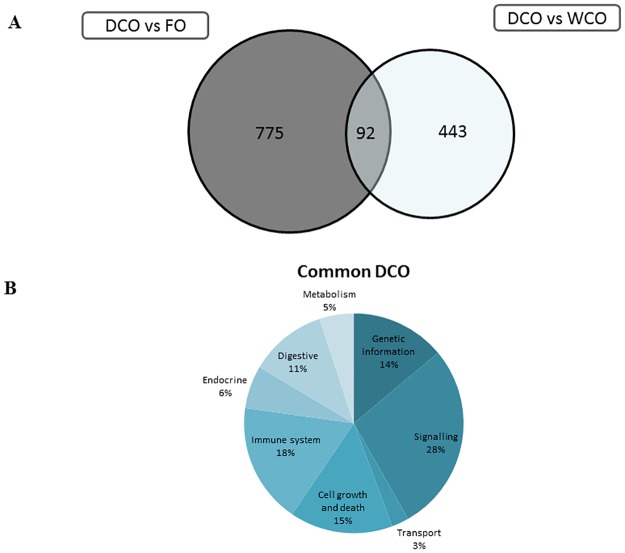
Impact of diet on hindgut transcriptome of Atlantic salmon fed diets containing Fish Oil (FO) or Wild type Camelina Oil (WCO) in comparison with fish fed fish EPA+DHA Camelina Oil (DCO). (A) Venn diagram representing transcripts differentially expressed in hindgut of Atlantic salmon fed diets FO and WCO compared to diet DCO. The area of the circles is scaled to the number of transcripts (Welch t-test; p < 0.05; FC > 1.3). (B) Distribution by categories of common differentially genes in hindgut between Atlantic salmon fed FO and WCO compared to DCO-fed fish (Welch t-test; p < 0.05; FC > 1.3). Non-annotated genes and features corresponding to the same gene are not represented.

There were 92 genes commonly differentially expressed (FC > 1.3, p < 0.005) when comparing the hindgut of DCO-fed with fish fed either FO or WCO (Fig 6). Good agreement was found in direction of expression between the two contrasts. KEGG analysis of these 92 DEG revealed that 56.5% of probes were annotated, resulting in 52 features representing 29 unique genes. The main category represented was signalling, followed by immune system and genetic information processing, but only three genes belonging to metabolism were commonly regulated in both contrasts and one of these, *dimethylaniline monooxygenase*, was also commonly regulated in the midgut (S5 Table).

The microarray data were validated by qPCR by comparing the expression of 6 different genes involved in metabolism in the midgut of fish fed DCO and WCO (S6 Table). Good correspondence in terms of FC and direction of change (up- or down-regulated) was observed among all the studied genes (100%). The match was also consistent in terms of significance (p value) when comparing qPCR and microarray results (83.3%; 5 out of 6 genes).

### Expression of key metabolic genes

**Fig 7:**
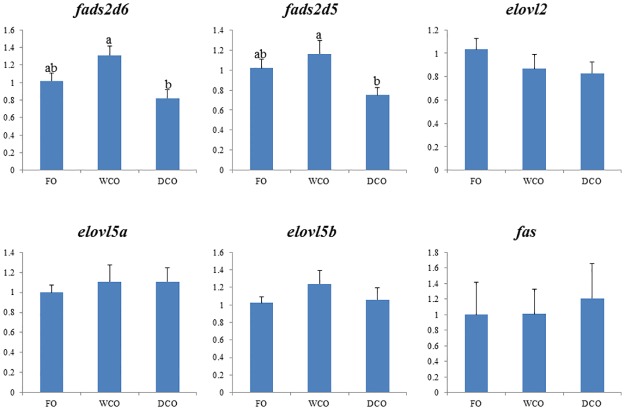
Expression of genes of the LC-PUFA biosynthesis pathway in liver of Atlantic salmon as determined by qPCR. Results are normalized expression ratios (means ± SEM; n = 6). FO, fish oil diet; WCO, wild type camelina oil diet; DCO, EPA+DHA camelina oil diet. *fads2d6*, delta-6 fatty acyl desaturase; *fads2d5*, delta-5 fatty acyl desaturase; *elovl2*, fatty acyl elongase 2; *elovl5a*, fatty acyl elongase 5 isoform a; *elovl5b*, fatty acyl elongase 5 isoform b; *fas*, fatty acid synthase. Different superscript letters denote differences among treatments identified by one-way ANOVA.

**Fig 8:**
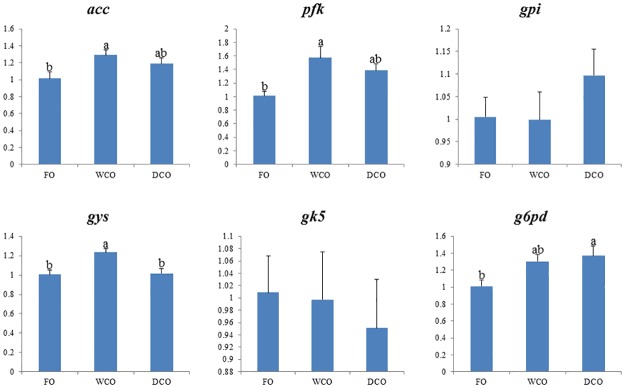
Expression of genes involved in lipogenesis and carbohydrate metabolism in liver of Atlantic salmon as determined by qPCR. Results are normalized expression ratios (means ± SEM; n = 6). FO, fish oil diet; WCO, wild type camelina oil diet; DCO, EPA+DHA camelina oil diet. *Fas*, fatty acid synthase; *acc*, acetyl CoA carboxylase; *gpi*, glucose-6-phosphate isomerase; *gys*, glycogen synthase; *pfk*, phosphofructokinase; *pk*, pyruvate kinase; *g6pd*, glucose-6-phosphate dehydrogenase; *gk5*, glycerol kinase 5.

To further focus on metabolic responses, the expression of key genes in either “candidate” pathways or pathways found to be differentially regulated by gut microarray was investigated by qPCR in both liver and intestinal tissues. Expression of delta-6 and delta-5 fatty acyl desaturases, *fads2d6* and *fads2d5*, was down-regulated (p = 0.008 and 0.038 respectively) in liver of fish fed DCO compared to fish fed WCO (Fig 7). There were no differences in hepatic expression of any of the fatty acid elongases evaluated. Relative expression of acetyl CoA carboxylase (*acc*), phosphofructokinase (*pfk*) and glycogen synthase (*gys*) was highest in liver of fish fed WCO, significantly so compared to fish fed FO with fish fed DCO displaying intermediate values (Fig 8). Expression of glucose-6-phosphate dehydrogenase (*g6pd*) was higher in liver of fish fed DCO than in liver of fish fed FO with fish fed WCO being intermediate. Other genes of lipogenesis (fatty acid synthase, *fas*) and carbohydrate metabolism (glucose-6-phosphate isomerase, *gpi*; glycerol kinase 5, *gk5*) showed no differences in liver expression between diets (Figs 7 and 8).

**Fig 9:**
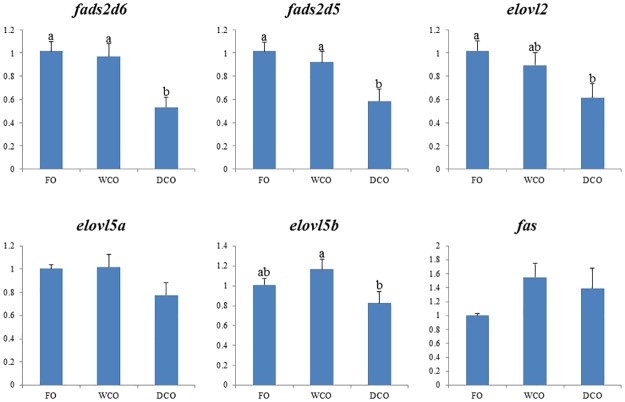
Expression of genes of the LC-PUFA biosynthesis pathway in midgut of Atlantic salmon as determined by qPCR. Results are normalized expression ratios (means ± SEM; n = 6). FO, fish oil diet; WCO, wild type camelina oil diet; DCO, EPA+DHA camelina oil diet. *fads2d6*, delta-6 fatty acyl desaturase; *fads2d5*, delta-5 fatty acyl desaturase; *elovl2*, fatty acyl elongase 2; *elovl5a*, fatty acyl elongase 5 isoform a; *elovl5b*, fatty acyl elongase 5 isoform b; *fas*, fatty acid synthase. Different superscript letters denote differences among treatments identified by one-way ANOVA.

**Fig 10:**
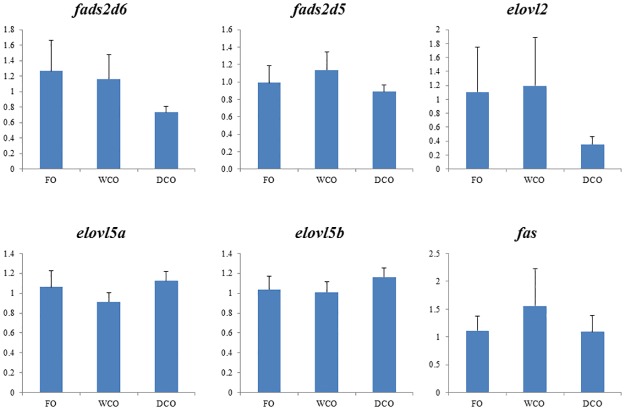
Expression of genes of the LC-PUFA biosynthesis pathway in hindgut of Atlantic salmon as determined by qPCR. Results are normalized expression ratios (means ± SEM; n = 6). FO, fish oil diet; WCO, wild type camelina oil diet; DCO, EPA+DHA camelina oil diet. *fads2d6*, delta-6 fatty acyl desaturase; *fads2d5*, delta-5 fatty acyl desaturase; *elovl2*, fatty acyl elongase 2; *elovl5a*, fatty acyl elongase 5 isoform a; *elovl5b*, fatty acyl elongase 5 isoform b; *fas*, fatty acid synthase. Different superscript letters denote differences among treatments identified by one-way ANOVA.

**Fig 11:**
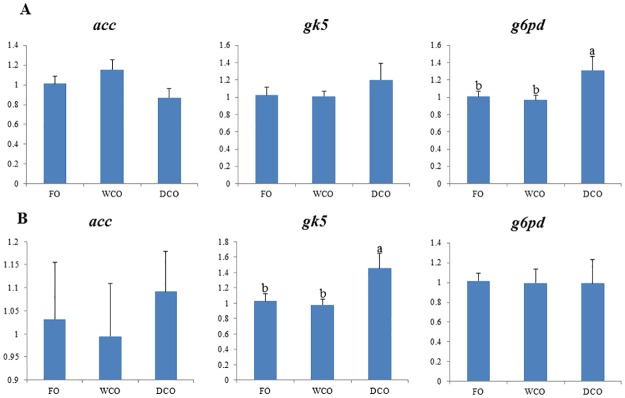
Expression of genes involved in lipogenesis and carbohydrate metabolism in Atlantic salmon midgut (A) and hindgut (B) as determined by qPCR. Results are normalized expression ratios (means ± SEM; n = 6). FO, fish oil diet; WCO, wild type camelina oil diet; DCO, EPA+DHA camelina oil diet. *Fas*, fatty acid synthase; *acc*, acetyl CoA carboxylase; *gpi*, glucose-6-phosphate isomerase; *gys*, glycogen synthase; *pfk*, phosphofructokinase; *pk*, pyruvate kinase; *g6pd*, glucose-6-phosphate dehydrogenase; *gk5*, glycerol kinase 5.

The expression of genes of LC-PUFA biosynthesis were down-regulated in midgut of salmon fed DCO, with *fads2d6* and *fads2d*5 expression in fish fed DCO being significantly lower than in fish fed FO or WCO (Fig 9). The same trend was observed for all the elongases with expression of *elovl2* and *elovl5b* being lower in midgut of fish fed DCO than fish fed FO or WCO, respectively. Similar trends in the genes of LC-PUFA biosynthesis were observed in hindgut, with lowest expression of *fads2d6*, *fads2d5* and *elovl2* all being lower in fish fed DCO than fish fed the other diets, albeit not significantly (Fig 10). Expression of *acc* in intestinal tissues was not affected by diet, whereas expression of *g6pd* in midgut (Fig 11A) and *gk5* in hindgut (Fig 11B) was higher in fish fed DCO than in fish fed either of the other diets.

### Detection of transgenic DNA in Atlantic salmon tissues

All salmon tissues tested negative for the presence of the camelina T-DNA gene construct, as monitored by the use of *DsRed* primers, directed towards the transgene construct sequence, whereas all tissues tested positive to salmon growth hormone gene, *gh* (data not shown).

## Discussion

Genetic modification of oilseed crops is a feasible option to produce entirely new sources of EPA and DHA that can replace FO in feeds without the negative effects on n-3 LC-PUFA content of farmed fish [8]. Previous studies using oils from transgenic *Camelina sativa* confirmed that the oils could maintain fish growth and survival at equivalent levels to fish fed a “gold standard” feed with high fishmeal and fish oil and, in addition, enhanced flesh n-3 LC-PUFA levels compared to fish fed conventional vegetable oil [11–14]. In the present study, a lower level of fishmeal (35% of diet) was used compared to the previous trials (49%) and EPA + DHA levels in the control (FO) diet were around 6% compared with > 25% in previous trials. Thus, the control diet in the present trial was more representative of commercial feeds, and close to the Norwegian standard of recent years [27–28].

The EPA+DHA oil from transgenic *Camelina* proved to be suitable for the on-growing of Atlantic salmon post-smolts as indicated by the good growth performance achieved by fish fed the DCO diet. In agreement with previous studies, no differences were found in terms of performance between DCO-fed fish and fish fed either FO or WCO [13–14]. Additionally, no adverse effects were observed in fish fed DCO on any of the plasma biochemistry parameters evaluated at the end of the feeding trial or 1 h and 24 h after the environmental stress test. Disturbance of osmoregulatory capacity is a characteristic response to stress in fish [29] and typical indicators of the stress response such as elevated blood cortisol, glucose, lactate, sodium, potassium and chloride were evident 1 h after stress in all diet groups, confirming the efficacy of the stress challenge. However, there was little effect of diet on the response of salmon to this particular stress test. Previous studies in gilthead sea bream (*Sparus aurata*) demonstrated that fish fed high levels of vegetable oil had higher levels of cortisol after stress and also the time for plasma cortisol to return basal levels was greater [30–31]. However, no differences due to diet were found among fish 1 h after being subjected to a chasing stress in Atlantic salmon fed high levels of both plant meal and vegetable oil [32], or in sunshine bass (*Morone chrysops x M*. *saxatilis*) fed increasing levels of plant meals after a low-water stress challenge [33]. This could indicate species-dependent responses to plant-based feeds and/or to different stressors. Furthermore, it may be necessary to evaluate responses at intermediate points (e.g. 4 h to 6 h), as differences in plasma biochemistry between fish fed marine- and plant-based diets have been reported in this threshold [31].

A major consequence of sustainable feeds based on plant ingredients is reduced levels of n-3 LC-PUFA in the flesh of farmed fish [8]. This has been demonstrated in many studies (see [34]), and the flesh n-3 LC-PUFA content of farmed salmon has been reported recently to have reduced by 50% over the last decade [9]. In the present study, the FO (control) feed was formulated to reflect recent feed formulations for salmon and thus included reduced levels of fishmeal and fish oil (replaced by rapeseed oil), which resulted in an n-3 LC-PUFA level of around 6% of total dietary fatty acids. Replacing the added oil with the oil from transgenic *Camelina* resulted in n-3 LC-PUFA content of the DCO diet being over 17% of total fatty acids that, in turn, doubled the n-3 LC-PUFA content of flesh of the DCO-fed fish compared to fish fed the current commercial formulation (diet FO). Thus, substituting the fish oil/vegetable oil blend of the commercial formulation by the new oil from transgenic *Camelina* has the potential to restore the n-3 LC-PUFA levels in harvest-sized salmon to levels last found a decade ago, retaining all the positive health effects associated with consumption of farmed salmon.

Salmonids have the capability to biosynthesize LC-PUFA with liver and midgut being the most active metabolic sites [35–36]. A common finding when Atlantic salmon are fed diets rich in vegetable oil (high C_18_ PUFA, zero n-3 LC-PUFA) is up-regulated expression of hepatic fatty acid desaturases and elongases, which in turn leads to increased production of EPA and DHA as well as intermediate products (i.e. 20:4n-3 and 22:5n-3) [11–12; 37–39]. Fish fed the FO and WCO diets, that had lower n-3 LC-PUFA contents than the DCO diet, showed up-regulation of hepatic *fads2d6* and *fad62d5* but, in the case of fish fed WCO, this was insufficient to compensate for the low levels of n-3 LC-PUFA in this diet. In contrast, although dietary n-3 LC-PUFA levels in diet FO were intermediate between those in WCO and DCO diets, the n-3 LC-PUFA level and, especially DHA, was surprisingly similar in liver of fish fed diets FO and DCO. The same patterns in gene expression and the proportions of DHA in liver were also observed in mid and hindgut tissues, other tissues with active LC-PUFA biosynthesis. Suppression of *fads2d6* expression by dietary DHA may be the main mechanism to inhibit n-3 LC-PUFA biosynthesis [40]. However, it was also suggested that activation of the LC-PUFA biosynthetic pathway in fish fed vegetable oil was due to both low dietary levels of the pathway products (EPA and DHA) as well as the presence of precursor (18:3n-3) [41]. Therefore, the impact of dietary fatty acids, other than pathway products EPA and DHA, on the expression of desaturases and elongases may be relevant and, indeed, the level of 18:3n-3 was one of the main differences between the three diets and this could be factor in the differences found between fish fed FO and WCO. It was shown previously that high levels of dietary 18:3n-3 are actually counter-productive for maintaining n-3 LC-PUFA levels in salmon fed linseed oil with a similar fatty acid composition to the camelina oil used in diet WCO [40]. The FO diet had the lowest level of 18:3n-3 and the highest 18:2n-6/18:3n-3 ratio, although high levels of this ratio did not inhibit DHA production in Atlantic salmon [41]. However, the precise mechanism for the surprisingly high levels of DHA in liver and intestinal tissues of FO-fed fish is unclear.

The intestine is not only the site of nutrient uptake, as the midgut also plays an active role in LC-PUFA biosynthesis [42–43], while hindgut is involved in the uptake and transport of antigens and final processing by intraepithelial macrophages [44]. In the present study, a greater number of genes were regulated in midgut than in hindgut, probably reflecting the role of midgut in digestion and nutrient metabolism. In general, all the diet contrasts presented the same gene categories in both gut regions, suggesting similar dietary effects despite their different physiological roles. The KO process with the highest number of DEG was amino acid metabolism in the DCO vs WCO contrast, particularly in midgut. However, within lipid metabolism, a high number of genes were down-regulated in the categories glycerolipid and glycerophospholipid metabolism in fish fed DCO compared to fish fed FO, in agreement with results found in the liver transcriptome previously [13]. In contrast, in gilthead sea bream liver and anterior intestine the expression of *lpcat1*, a key player in phospholipid remodelling, showed up-regulation or no regulation in fish fed DCO compared to FO-fed fish [14]. Although several studies have found that dietary n-3 PUFA can regulate the expression of this enzyme in teleosts [43–47], differing results in terms of direction of regulation were observed, indicating species differences.

Another pathway that was significantly regulated in both intestinal tissues was Cytokine-Cytokine receptor interaction, particularly in hindgut in the FO-WCO contrast. Altered immune system in teleosts in response to dietary vegetable oils was first reported in Atlantic salmon [48] and since then a number of studies have reported adverse effects [49–50]. Thus, regulation in expression of several cytokines was reported previously in the posterior intestine of sea bream [14] and sea bass [51] fed high levels of vegetable oil. However, it is important to note that in the present study these pathways were more affected in fish fed WCO than fish fed DCO. Similarly, the up-regulation in several immune pathways related to apoptosis, T cell receptor or leukocyte transendothelial migration in the hindgut of fish fed WCO compared to FO-fed fish was not so pronounced in fish fed DCO. All the diets in the present study contained similar levels of pro-inflammatory n-6 PUFA and so it was most likely the dietary level of n-3 LC-PUFA that influenced these effects on immune pathways with fish fed WCO, with the lowest level of anti-inflammatory EPA and DHA, showing the greatest impact on intestinal health. However, most studies evaluating sustainable feeds have focussed on the replacement of fish meal by plant meals [52–53], whereas substitution of dietary lipid had had less attention. In the present trial no adverse effects were observed on either the mid or hindgut histology. Shorter folds in the midgut of salmon fed 80% vegetable oil were observed previously, although no other differences in histology, immunohistochemistry or expression of immune related genes were reported [54]. In agreement, no histological alterations were observed in intestine of sea bass when fed 60–70% vegetable oil [55–56]. Furthermore, previous studies using an EPA only oil from transgenic *Camelina* showed no intestinal alterations in either Atlantic salmon or sea bream [12,14].

Histopathological evaluation of the liver showed enhanced intra-cytoplasmic lipid deposition in WCO-fed fish (Table 7), which was associated with increased lipid accumulation in this tissue (Table 3) despite the lipid content of the WCO feed being slightly lower than the FO diet. Previous studies have reported the tendency of teleosts to accumulate lipid in liver when vegetable oils are included in feeds [11, 14; 57–58]. Concomitantly, up-regulation of the adipogenic enzyme *acc* was observed in WCO-fed fish, suggesting that the high levels of C_18_ fatty acids and reduced levels of n-3 LC-PUFA enhanced the synthesis of lipids, which in turn leads to hepatic lipid accumulation. Indeed, in mammals PUFA are known to be potent inhibitors of *de novo* lipogenesis through the inhibition of *acc* [59]. Importantly, in the present study, liver lipid level in fish fed DCO was restored to that of fish fed the FO diet

In the present study, reduced glycogen deposition in the liver of fish fed WCO and, especially DCO, was observed. Increased liver glycogen stores could indicate a positive energy balance [60–61] whereas reduced contents have been related to stress response [62]. In mammals, it has been shown that n-3 LC-PUFA stimulate glycogen synthesis [63] and also regulate some proteins involved in carbohydrate metabolism [64]. However, there are differences in nutrient use between salmon and mammals, such as poor use of dietary glucose [60], and thus the effects of n-3 LC-PUFA on carbohydrate metabolism in salmon are unclear. In the present trial, DCO increased expression of *g6pd* consistent with previous reports using sustainable feeds in sea bass [65]. The *g6pd* enzyme catalyzes NADP-linked oxidation of glucose-6-phosphate, and is a major provider of NADPH for lipogenesis in salmon [66]. Given that L cholesterol biosynthesis require reducing power in the form of NADPH, increased expression of *g6pd* may be related to lower levels of cholesterol in the feeds. Fish fed WCO showed the highest expression of the glycolytic enzyme *pfk*. Similarly, low dietary fish oil enhanced the activity of *pfk* in the muscle of Senegalese sole [67], which could suggest the utilization of stored energy due to the fish being in an “adverse” nutritional situation. In contrast, no effect on hepatic expression or activity of *pfk* was found in rainbow trout fed diets with or without fish oil, these fish also showing limited effects on enzymes involved in lipogenesis, fatty acid β-oxidation or amino acid oxidation [68]. However, rainbow trout in fresh water likely have a higher capacity to synthesize n-3 LC-PUFA and, therefore, low dietary requirements for LC-PUFA, which could explain the reduced response compared to marine teleosts [69]. The highest expression of glycogen synthase (*gys*) was also observed in WCO-fed fish, which may be a compensatory mechanism to the increased use of glycogen as indicated by the high expression of *pfk*. Synthesis of hepatic glycogen by glycogen synthase utilizes UDP-glucose as one substrate and the non-reducing end of glycogen as another. *De novo* gluconeogenesis, probably from dietary amino acids, has been shown in carnivorous marine fish species fed fishmeal-based (low carbohydrate) diets after starvation [70]. Furthermore, a direct relationship between hepatocyte glycogen and glycogen synthase activity was observed in rainbow trout hepatocytes incubated with glucose indicating that the direct pathway for glycogen synthesis was active [71]. However, hepatocyte glycogen content may be controlled by several factors, including n-3 LC-PUFA levels. In this respect, feeding rats a diet containing n-3 LC-PUFA significantly inhibited pyruvate kinase, a key glycolytic enzyme [72].

To conclude, the oil from transgenic *Camelina sativa* containing EPA and DHA effectively substituted for fish oil in feeds for Atlantic salmon, supporting good growth without compromising fish health. No adverse effects were observed on plasma biochemistry or intestinal transcriptomes, and intestinal histology was normal. Furthermore, compared to fish fed diet FO, with an n-3 LC-PUFA level similar to commercial feeds, DCO-fed fish accumulated much higher levels of these beneficial fatty acids. Thus, the use of this entirely new source of EPA and DHA could help to maintain or even boost the n-3 LC-PUFA in aquaculture produce without compromising the sustainability of the feeds.

## Supporting information

S1 Table Fatty acid compositions (percentage of fatty acids) of the four oils used in the present trial. (DOCX)

S2 Table Primer sequences used for qPCR or PCR analysis. (DOCX)

S3 Table Description of the semi-quantitative scoring system using different parameters to assess the liver, mid gut and hind gut of Atlantic salmon fed the three experimental feeds containing different lipid sources. The presence of glycogen and Goblet cells was assessed using PAS staining, whereas the other parameters were scored with H&E staining.
(DOCX)

S4 Table Annotated transcripts within the 122 features exhibiting common differential expression in midgut of Atlantic salmon fed DCO compared to fish fed either FO or WCO diets. Features are arranged by functional categories and within them by increasing p value (assessed by Welch t-test).
(DOCX)

S5 Table Annotated transcripts in the 92 features exhibiting common differential expression in hind gut of Atlantic salmon fed DCO compared to fish fed either FO or WCO diets. Features are arranged by functional categories and within them by increasing p value (assessed by Welch t-test).
(DOCX)

S6 Table Validation of microarray results by qPCR. (DOCX)

S1 Fig Absolute n-3 LC-PUFA contents (mg) per portion of fillet (130 g). n-3 LC-PUFA, omega-3 long chain polyunsaturated fatty acids (sum of 20:4n-3, 20:5n-3, 22:5n-3 and 22:6n-3).
(DOCX)

## References

[1] Campoy C, Escolano-Margarit V, Anjos T, Szajewska H, Uauy R. Omega 3 fatty acids on child growth, visual acuity and neurodevelopment. Br J Nutr. 2012; 107: S85–S106 10.1017/S0007114512001493 22591907

[2] Delgado-Lista J, Perez-Martinez P, Lopez-Miranda J, Perez-Jimenez F. Long chain omega-3 fatty acids and cardiovascular disease: a systematic review. Br J Nutr. 2012; 107: S201–S213. 10.1017/S0007114512001596 22591894

[3] Gil A, Serra-Majem L, Calder PC, Uauy R. Systematic reviews of the role of omega-3 fatty acids in the prevention and treatment of disease. Br J Nutr. 2012; 107: S1–S2 10.1017/S0007114512001420 22591884

[4] Global Organisation for EPA and DHA (GOED), Global recommendations for EPA and DHA intake. 19 November 2014. http://issfal.org/GlobalRecommendationsSummary19Nov2014Landscape_-3-.pdf

[5] American Heart Association (AHA), Fish 101. 2015. http://www.heart.org/HEARTORG/GettingHealthy/NutritionCenter/Fish-101_UCM_305986_Article.jsp#aha_recommendation

[6] Harwood JL, Guschina IA. The versatility of algae and their lipid metabolism. Biochimie. 2009; 91: 679–684. 10.1016/j.biochi.2008.11.004 19063932

[7] FAO. State of World Fisheries and Aquaculture 2016. Rome: Food and Agriculture Organization of the United Nations; 2016.

[8] Tocher DR. Omega-3 long-chain polyunsaturated fatty acids and aquaculture in perspective. Aquaculture. 2015; 449: 94–107.

[9] Sprague M, Dick JR, Tocher DR. Impact of sustainable feeds on omega-3 long-chain fatty acid levels in farmed Atlantic salmon, 2006–2015. Sci Rep. 2016; 21892. 10.1038/srep21892 PMC4761991 26899924

[10] Ruiz-Lopez N, Haslam RP, Napier JA, Sayanova O. Successful high-level accumulation of fish oil omega-3 long-chain polyunsaturated fatty acids in a transgenic oilseed crop. Plant J. 2014; 77: 198–208. 10.1111/tpj.12378 24308505 PMC4253037

[11] Betancor MB, Sprague M, Usher S, Sayanova O, Campbell PJ, Napier JA, et al A nutritionally-enhanced oil from transgenic *Camelina sativa* effectively replaces fish oil as a source of eicosapentaenoic acid for fish. Sci Rep. 2015; 5: 8104 10.1038/srep08104 25632018 PMC4309969

[12] Betancor MB, Sprague M, Sayanova O, Usher S, Campbell PJ, Napier JA, et al Evaluation of a high-EPA oil from transgenic *Camelina sativa* in feeds for Atlantic salmon (*Salmo salar* L.): Effects on tissue fatty acid composition, histology and gene expression. Aquaculture. 2015; 444: 1–12. 10.1016/j.aquaculture.2015.03.020 26146421 PMC4459488

[13] Betancor MB, Sprague M, Sayanova O, Usher S, Metochis C, Campbell PJ, et al Nutritional evaluation of an EPA-DHA oil from transgenic *Camelina sativa* in feeds for post-smolt Atlantic salmon (*Salmo salar* L.). PLoS ONE. 2016; 11: e0159934 10.1371/journal.pone.0159934 27454884 PMC4959691

[14] Betancor MB, Sprague M, Montero D, Usher S, Sayanova O, Campbell PJ, et al Replacement of marine fish oil with de novo omega-3 oils from transgenic *Camelina sativa* in feeds for gilthead sea bream (*Sparus aurata*). Lipids. 2016; 51: 1171–1191. 10.1007/s11745-016-4191-4 27590240 PMC5418318

[15] Tejera N, Vauzour D, Betancor MB, Sayanova O, Usher S, Cochard M, et al A transgenic *Camelina sativa* seed oil replaces fish oil as a dietary source of EPA in mice. J Nutr. 2016; 146: 227–235. 10.3945/jn.115.223941 26791554 PMC4725436

[16] AOAC. Official Methods of Analysis. Washington, DC: Association of Official Analytical Chemists; 2000.

[17] Folch J, Lees N, Sloane-Stanley GH. A simple method for the isolation and purification of total lipids from animal tissues. J Biol Chem. 1957; 226: 497–509. 13428781

[18] Christie WW. Lipid Analysis. 3^rd^ ed Bridgwater: Oily Press; 2003.

[19] Ackman RG, Eaton CA. Some contemporary applications of opem-tubular gas liquid chromatography in analyses of methyl esters of longer-chain fatty acids. Eur J Lipid Sci Technol. 1978; 80: 21–37.

[20] Betancor MB, Howarth FJE, Glencross BD, Tocher DR. Influence of dietary docosahexaenoic acid in combination with other long-chain polyunsaturated fatty acids on expression of biosynthesis genes and phospholipid fatty acid compositions in tissues of post-smolt Atlantic salmon (*Salmo salar*). Comp Biochem Physiol B. 2014; 172–173: 74–89. 10.1016/j.cbpb.2014.04.007 24807616

[21] Rozen S, Skaletsky H. Primer3 on the WWW for general users and for biologist programmers. Methods Mol Biol. 2000; 132: 365–386 10547847 10.1385/1-59259-192-2:365

[22] Uran PA, Goncalvez AA, Taverme-Thielem JJ, Schrama JW, Verreth JAJ, Rombout JHWM. Soybean meal induces intestinal inflammation in common carp (*Cyprinus carpio* L.). Fish Shellfish Immunol. 2008; 25: 751–760. 10.1016/j.fsi.2008.02.013 18954997

[23] McFadzen IRB, Coombs SH, Halliday NC. Histological indices of the nutritional condition of sardine, *Sardina pilchardus* (Walbaum) larvae off the north coast of Spain. J Exp Mar Biol Ecol. 1997; 212: 239–258.

[24] Demissie M. Mascialino B, Calza S, Pawitan Y. Unequal group variances in microarray data analyses. Bioinformatics. 2008; 24: 1168–1174. 10.1093/bioinformatics/btn100 18344518

[25] Kanehisa M, Goto S. KEGG: Kyoto encyclopedia of genes and genomes. Nucleic Acid Res. 2000; 28: 27–30. 10592173 10.1093/nar/28.1.27 PMC102409

[26] Pfaffl MW, Morgan GW, Dempfle L. Relative expression software tool (REST) for group-wise comparison and statistical analysis of relative expression results in real-time PCR. Nucleic Acids Res. 2002; 30: e36 11972351 10.1093/nar/30.9.e36 PMC113859

[27] Ytrestøyl T, Aas TS, Åsgård T. Utilisation of feed resources in production of Atlantic salmon (*Salmo salar*) in Norway. Aquaculture. 2015; 448: 365–374.

[28] Shepherd CJ, Monroig Ó, Tocher DR. Future availability of Scottish salmon feeds and supply chain implications. Aquaculture. 2017; 467: 49–62.

[29] Bonga SEW. The stress response in fish. Physiol Rev. 1997; 77: 591–625. 9234959 10.1152/physrev.1997.77.3.591

[30] Ganga R, Montero D, Bell JG, Atalah E, Ganuza E, Vega-Orellana O, et al Stress response in sea bream (*Sparus aurata*) held under crowded conditions and fed diets containing linseed or soybean oil. Aquaculture. 2011; 311: 215–223.

[31] Pérez-Sánchez J, Borrel M, Bermejo-Nogales A, Benedito-Palos L, Saera-Vila A, Calduch-Giner JA, et al Dietary oils mediate cortisol kinetics and the hepatic mRNA expression profile of stress-responsive genes in gilthead sea bream (*Sparus aurata*) exposed to crowding stress. Implications on energy homeostasis and stress susceptibility. Comp Biochem Physiol. 2013; 8D: 123–130. 10.1016/j.cbd.2013.02.001 23466468

[32] Oxley A, Jolly C, Eide T, Jordal AEO, Svardal A, Olsen RE. The combined impact of plant-derived dietary ingredients and acute stress on the intestinal arachidonic cascade in Atlantic salmon (*Salmo salar*). Br J Nutr. 2009; 103: 851–861. 10.1017/S0007114509992467 19943982

[33] Laporte J, Trushenki J. Production performance, stress tolerance and intestinal integrity of sunshine bass fed increasing levels of soybean meal. J Anim Physiol Anim Nutr. 2011; 96: 513–526. 10.1111/j.1439-0396.2011.01174.x 21651622

[34] Turchini GM, Ng W-K, Tocher DR. (2011) Fish Oil Replacement and Alternative Lipid Sources in Aquaculture Feeds. Boca Raton: Taylor & Francis, CRC Press; 2011.

[35] Morais S, Monroig O, Zheng X, Leaver MJ, Tocher DR. Highly unsaturated fatty acid synthesis in Atlantic salmon: Characterization of ELOVL5- and ELOVL2- like elongases. Mar Biotechnol. 2009; 11: 627–639. 10.1007/s10126-009-9179-0 19184219

[36] Zheng X, Leaver MJ, Tocher DR. Long-chain polyunsaturated fatty acid synthesis in fish: Comparative analysis of Atlantic salmon (*Salmo salar* L.) and Atlantic cod (*Gadus morhua* L.) Δ6 fatty acyl desaturase gene promoters. Comp Biochem Physiol. 2009; 154B: 255–263. 10.1016/j.cbpb.2009.06.010 19563904

[37] Leaver MJ, Bautista JM, Björnsson T, Jönsson E, Krey G, Tocher DR et al Towards fish lipid nutrigenomics: current state and prospects for fin-fish aquaculture. Rev Fisheries Sci. 2008; 16: 71–92.

[38] Bell MV, Tocher DR. Biosynthesis of fatty acids; general principles and new directions In: Arts MT, Brett M, Kainz M, editors. Lipids in Aquatic Ecosystems. New York: Springer-Verlagpp. 2009 pp. 211–236.

[39] Torstensen BE, Tocher DR (2010) The Effects of fish oil replacement on lipid metabolism of fish In: Turchini GM, Ng W-K, Tocher DR, editors. Fish Oil Replacement and Alternative Lipid Sources in Aquaculture Feeds. Boca Raton: Taylor & Francis, CRC Press; 2011. pp.405–437.

[40] Tocher DR, Fonseca-Madrigal J, Bell JG, Dick JR, Henderson RJ, Sargent JR. Effects of diets containing linseed oil on fatty acid desaturation and oxidation in hepatocytes and intestinal enterocytes in Atlantic salmon (*Salmo salar*). Fish Physiol. Biochem. 2002; 26: 157–170.

[41] Sissener NH, Sanden M, Torstensen BE, Waagbø R, Stubhaug I, Rosenlund G. High dietary 18:2n-6/18:3n-3 ratios does not inhibit elongation and desaturation of 18:3n-3 to EPA and DHA in Atlantic salmon (*Salmo salar* L.). Aquacult Nutr. Forthcoming.

[42] Tocher DR, Zheng X, Schlechtriem C, Hastings N, Dick JR, Teale AJ. Highly unsaturated fatty acid synthesis in marine fish: Cloning, functional characterization and nutritional regulation of fatty acyl Δ6 desaturase of Atlantic cod (*Gadus morhua* L.). Lipids. 2006; 41: 1003–1016. 17263300 10.1007/s11745-006-5051-4

[43] Vagner M, Santigosa E. Characterization and modulation of gene expression and enzymatic activity of delta-6-desaturase in teleost: a review. Aquaculture. 2011; 315: 131–143.

[44] Rombout JHWM, Van den Berg AA. Immunological importance of the second gut segment of carp. I. Uptake and processing of antigens by epithelial cells and macrophages. J Fish Biol. 1989; 35: 13–22.

[45] Benedito-Palos L, Calduch-Giner JA, Ballester-Lozano GF, Pérez-Sánchez J. Effect of ration size on fillet fatty acid compositions, phospholipid allostasis and mRNA expression patterns of lipid regulatory genes in gilthead sea bream (*Sparus aurata*). Br J Nutr. 2013; 109: 1175–1187. 10.1017/S000711451200311X 22856503

[46] Rimoldi S, Benedito-Palos L, Terova G, Pérez-Sánchez J. Wide-targeted gene expression infers tissue-specific molecular signatures of lipid metabolism in fed and fasted fish. Rev Fish Biol Fisheries. 2015; 26: 93–108.

[47] Bonacic K, Campoverde C, Sastre M, Hachero-Cruzado I, Ponce M, Manchado M, et al Mechanisms of lipid metabolism and transport underlying superior performance of Senegalese sole (*Solea senegalensis*, Kaup 1858) larvae fed diets containing n-3 polyunsaturated fatty acids. Aquaculture. 2016; 450: 383–396.

[48] Bransden MP, Carter CG, Nichols PD. Replacement of fish oil with sunflower oil in feeds for Atlantic salmon (*Salmo salar* L.): effect on growth performance, tissue fatty acid composition and disease resistance. Comp Biochem Physiol. 2003; 135B: 611–625. 10.1016/s1096-4959(03)00143-x 12892753

[49] Montero D, Grasso V, Izquierdo MS, Ganga R, Real E, Tort L, et al Total substitution of fish oil by vegetable oils in gilthead sea bream (*Sparus aurata*) diets: effects on hepatic Mx expression and some immune parameters. Fish Shellfish Immunol. 2008; 24: 147–155. 10.1016/j.fsi.2007.08.002 18158252

[50] Kiron V, Thawonsuwan J, Panigrahi A, Scharsack JP, Satoh S. Antioxidant and immune defences of rainbow trout (*Oncorhynchus mykiss*) offered plant oils differing in fatty acid profiles from early stages. Aquacult Nutr. 2011; 17: 130–140.

[51] Torrecillas S, Montero D, Caballero MJ, Pittman KA, Custódio M, Campo A, et al Dietary mannan oligosaccharides: counteracting the side effects of soybean meal oil inclusion on European sea bass (*Dicentrarchus labrax*) gut health and skin mucosa mucus production? Front Immunol. 2015; 6: 397 10.3389/fimmu.2015.00397 26300883 PMC4525062

[52] Bæverfjord G, Krogdahl Å. Development and regression of soybean meal induced enteritis in Atlantic salmon, *Salmo salar* L., distal intestine: a comparison with the intestines of fasted fish. J Fish Dis. 1996; 19: 375–387.

[53] Penn MH, Bendiksen EA, Campbell P, Krogdahl Å. High level of dietary pea protein concentrate induces enteropathy in Atlantic salmon (*Salmo salar* L.). Aquaculture. 2011; 310: 267–273.

[54] Moldal T, Løkka G, Wiik-Nielsen J, Austbø L, Torstensen BE, Rosenlund G, et al Substitution of dietary fish oil with plant oils is associated with shortened mid intestinal folds in Atlantic salmon (*Salmo salar*). BMC Vet Res. 2014; 10: 60 10.1186/1746-6148-10-60 24606841 PMC3973862

[55] Mourente G, Good JE, Thompson KD, Bell JG. Effects of partial substitution of dietary fish oil with blends of vegetable oils on blood leucocyte fatty acid compositions, immune function and histology in European sea bass (*Dicentrarchus labrax*). Br J Nutr. 2007; 98: 770–779. 10.1017/S000711450773461X 17466094

[56] Castro C, Couto A, Pérez-Jiménez A, Serra CR, Díaz-Rosales P, Fernandes R, et al Effects of fish oil replacement by vegetable oil blend on digestive enzymes and tissue histomorphology of European sea bass (*Dicentrarchus labrax*) juveniles. Fish Physiol Biochem. 2016; 42: 203–217. 10.1007/s10695-015-0130-1 26364216

[57] Fountoulaki E, Vasilaki A, Hurtado R, Grigorakis K, Karacostas I, Nengas I, et al Fish oil substitution by vegetable oils in commercial diets for gilthead sea bream (*Sparus aurata* L.); effects on growth performance, flesh quality and fillet fatty acid profile: Recovery of fatty acid profiles by a fish oil finishing diet under fluctuating water temperatures. Aquaculture. 2009; 289: 317–326.

[58] Peng M, Xu W, Mai K, Zhou H, Zhang Y, Liufu Z, et al Growth performance, lipid deposition and hepatic lipid metabolism related gene expression in juvenile turbot (*Scophtalmus maximus* L.) fed diets with various fish oil substitution levels by soybean oil. Aquaculture. 2014; 433: 442–449.

[59] Dentin R, Benhamed F, Pégorier JP, Foufelle F, Viollet B, Vaulont S, et al Polyunsaturated fatty acids suppress glycolytic and lipogenic genes through the inhibition of ChREBP nuclear protein translocation. J Clin Invest. 2005; 115: 2843–2854. 10.1172/JCI25256 16184193 PMC1224299

[60] Hemre GI, Mommsen TP, Krogdahl A. Carbohydrates in fish nutrition: effects on growth, glucose metabolism and hepatic enzymes. Aquacult Nutr. 2022; 8: 175–194.

[61] Ekman KS, Dalsgaard J, Holm J, Campbell PJ, Skov PV. Glycogenesis and de novo lipid synthesis from dietary starch in juvenile gilthead sea bream (*Sparus aurata*) quantified with stable isotopes. Br J Nutr. 2013; 109: 2135–2146. 10.1017/S000711451200445X 23186693

[62] Bleau H, Daniel C, Chevalier G, van Tra H, Hontela A. Effects of acute exposure to mercury chloride and methylmercury on plasma cortisol, T3, T4, glucose and liver glycogen in rainbow trout (*Oncorhynchus mykiss*). Aquat Toxicol. 1996; 34: 221–235.

[63] Kuda O, Jelenik T, Jilkova Z, Flachs P, Rossmeisl M, Hensler M, et al n-3 fatty acids and rosiglitazone improve insulin sensitivity through additive stimulatory effects on muscle glycogen synthesis in mice fed a high-fat diet. Diabetologia. 2009; 52: 941–951. 10.1007/s00125-009-1305-z 19277604

[64] Ahmed AA, Balogun KA, Bykova NV, Cheema SK. Novel regulatory roles of omega-3 fatty acids in metabolic pathways: a proteomics approach. Nutr Metab. 2014; 11: 6. 10.1186/1743-7075-11-6 PMC3898484 24438320

[65] Geay F, Ferraresso S, Zambonino-Infante JL, Bargelloni L, Quentel C, Vandeputte M, et al Effects of the total replacement of fish-based diet with plant-based diet on the hepatic transcriptome of two European sea bass (*Dicentrarchus labrax*) half-sibfamilies showing different growth rates with the plant-based diet. BMC Genom. 2011; 12: 522. 10.1186/1471-2164-12-522 PMC3377934 22017880

[66] Dias J, Huelvan C, Dinis MT, Metailler R. Influence of dietary bulk agents (silica, cellulose and a natural zeolite) on protein digestibility, growth, feed intake and feed transit time in European seabass (*Dicentrarchus labrax*) juveniles. Aquat Living Resour. 1998; 11: 219–226.

[67] Borges P, Medale F, Dias J, Valente LMP. Protein utilisation and intermediary metabolism of Senegalese sole (*Solea senegalensis*) as a function of protein:lipid ratio. Br J Nutr. 2013; 109: 1373–1381. 10.1017/S0007114512003418 22906759

[68] Ducasse-Cabanot S, Zambonino-Infante J, Richard N, Medale F, Corraze G, Mambrini M, et al Reduced lipid intake leafs to changes in digestive enzymes in the intestine but has minor effects on key enzymes of hepatic intermediary metabolism in rainbow trout (*Oncorhynchus mykiss*). Animal. 2007; 1: 1272–1282. 10.1017/S1751731107000596 22444883

[69] Tocher DR. Fatty acid requirements in ontogeny of marine and freshwater fish. Aquacult Res. 2010; 41: 717–732.

[70] Viegas I, Rito J, Jarak I, Leston S, Carvalho RA, Metón I, et al Hepatic glycogen synthesis in farmed European sea bass (*Dicentrarchus labrax* L.) is dominated by indirect pathway fluxes. Comp Biochem Physio. 2012; 163A: 22–29. 10.1016/j.cbpa.2012.04.023 22561667

[71] Pereira C, Vijayan MM, Storey KB, Jones RA, Moon TW. Role of glucose and insulin in regulating glycogen synthase and phosphorylase activities in rainbow trout hepatocytes. J Comp Physiol B. 1995; 165: 62–70.

[72] Yilmaz HR, Songur A, Özyurt B, Zararsiz I, Sarsilmaz M. The effects of n-3 polyunsaturated fatty acids by gavage on some metabolic enzymes of rat liver. Prostaglandins, Leukot Essent Fatty acids. 2004; 71: 131–135. 15207530 10.1016/j.plefa.2004.03.002

