# Supplementary material for: An oil containing EPA and DHA from transgenic Camelina sativa to replace marine fish oil in feeds for Atlantic salmon (Salmo salar L.): Effects on intestinal transcriptome, histology, tissue fatty acid profiles and plasma biochemistry
Source: PLoS One. 2017 Apr 12;12(4):e0175415. doi: 10.1371/journal.pone.0175415 (PMC5389825; doi:10.1371/journal.pone.0175415)
Supplement: S1 Table — (DOCX) [file pone.0175415.s001.docx]

**Supplementary Table 1**

|  | **Oils** | | | |
| --- | --- | --- | --- | --- |
|  | **Fish** | **Wt-CO** | **RO** | **Tr-CO** |
|  |  |  |  |  |
| Total saturated^1^ | 25.5 | 9.9 | 7.7 | 15.5 |
| Total monoenes^2^ | 40.9 | 36.4 | 67.5 | 19.9 |
| 18:2n-6 | 1.3 | 19.3 | 16.4 | 20.9 |
| 20:2n-6 | 0.2 | 1.5 | 0.1 | 1.3 |
| 20:3n-6 | 0.2 | n.d. | n.d. | 1.3 |
| 20:4n-6 | 0.6 | n.d. | n.d. | 2.1 |
| Total n-6 PUFA^3^ | 2.7 | 20.9 | 16.5 | 30.7 |
| 18:3n-3 | 1.0 | 31.9 | 8.3 | 13.0 |
| 20:3n-3 | 0.1 | 1.0 | n.d. | 0.8 |
| 20:4n-3 | n.d. | n.d. | n.d. | 2.3 |
| 20:5n-3 | 11.6 | n.d. | n.d. | 6.0 |
| 22:5n-3 | 1.3 | n.d. | n.d. | 1.5 |
| 22:6n-3 | 9.8 | n.d. | n.d. | 5.1 |
| Total n-3 PUFA^4^ | 28.0 | 32.9 | 8.3 | 32.7 |
| Total PUFA | 33.6 | 53.7 | 24.8 | 63.4 |
| Total n-3 LC-PUFA | 22.8 | n.d. | n.d. | 14.9 |

^1^Contains 14:0, 16:0, 18:0, 20:0, 22:0 and 24:0; ^2^Contains 16:1n-7, 18:1n-9, 18:1n-7, 20:1n-9, 22:1n-11 and 22:1n-9; ^3^Contains 18:2n-6; ^4^Contains 18:4n-3. LC-PUFA, long-chain polyunsaturated fatty acids (sum of 20:4n-3, 20:5n-3 22:5n-3 and 22:6n-3). n.d., not detected; RO, rapeseed oil; Tr-CO, oil from transgenic Camelina; Wt-CO, wild-type Camelina oil.
