# Supplementary material for: An oil containing EPA and DHA from transgenic Camelina sativa to replace marine fish oil in feeds for Atlantic salmon (Salmo salar L.): Effects on intestinal transcriptome, histology, tissue fatty acid profiles and plasma biochemistry
Source: PLoS One. 2017 Apr 12;12(4):e0175415. doi: 10.1371/journal.pone.0175415 (PMC5389825; doi:10.1371/journal.pone.0175415)
Supplement: S2 Table — (DOCX) [file pone.0175415.s002.docx]

**Supplementary Table 2**. Primer sequences used for qPCR or PCR analysis

| Aim | Transcript | Primer sequence (5’→3’) | Amplicon (bp) | Ta | Accession no | Probe size (bp) |
| --- | --- | --- | --- | --- | --- | --- |
| *qPCR* | *fads2d6* | F: TCCTCTGGTGCGTACTTTGT | 163 | 59˚C | NM_001123575.2 | 60 |
|  |  | R: AAATCCCGTCCAGAGTCAGG |  |  |  |  |
|  | *fads2d5* | F: GCCACTGGTTTGTATGGGTG | 148 | 59˚C | NM_001123542.2 | 60 |
|  |  | R: TTGAGGTGTCCACTGAACCA |  |  |  |  |
|  | *elovl2* | F: GGTGCTGTGGTGGTACTACT | 190 | 59˚C | NM_001136553.1 | 60 |
|  |  | R: ACTGTTAAGAGTCGGCCCAA |  |  |  |  |
|  | *elovl5a* | F: TGTTGCTTCATTGAATGGCCA | 150 | 59˚C | GU238431.1 | 60 |
|  |  | R: TCCCATCTCTCCTAGCGACA |  |  |  |  |
|  | *elovl5b* | F: CTGTGCAGTCATTTGGCCAT | 192 | 59˚C | NM_001136552.1 | 60 |
|  |  | R: GGTGTCACCCCATTTGCATG |  |  |  |  |
|  | *fas* | F: ACCGCCAAGCTCAGTGTGC | 212 | 60˚C | CK876943 | - |
|  |  | R: CAGGCCCCAAAGGAGTAGC |  |  |  |  |
|  | *gpi* | F: ACTTCCTCATTCCTGCCCAA | 191 | 60˚C | XM_014125588.1 | - |
|  |  | R: GAAGACTTTGTGTGGCAGCA |  |  |  |  |
|  | *gys* | F: GATCCATGGTTGCCTGGTTC | 170 | 59˚C | XM_014134465.1 | - |
|  |  | R: TCTTCCCAACAGAGTGGCAT |  |  |  |  |
|  | *acc* | F: GGATTGCCTGTATCTTGGAC | 92 | 59˚C | DW573070 | - |
|  |  | R: CTGGACGATACTCTGAGTGTTC |  |  |  |  |
|  | *pfk* | F: AATCCATCGGCGTTCTGACAAGC | 61 | 59˚C | BT059256 | - |
|  |  | R:GCCCGTACAGCAGCATTCATACCTT |  |  |  |  |
|  | *pk* | F: TGCCTTCATTCAGACGCAGCA | 117 | 59˚C | BT043851 | - |
|  |  | R: CAGATGATTCCGGTGTTGCGA |  |  |  |  |
|  | *g6pd* | F: TACCACGACGTCACCAAGAA | 171 | 59˚C | NM_001141724.1 | - |
|  |  | R: CAGGTAGTGGTCTATGCGGT |  |  |  |  |
|  | *gk5* | F: TGTTTGGTGAGTGCTGCTTC | 208 | 59˚C | XM_014189232.1 | - |
|  |  | R: AGCTCCTGTGCCCACTTAAT |  |  |  |  |
|  | *hprtq* | F: CCGCCTCAAGAGCTACTGTAAT | 256 | 55˚C | BT043501 | - |
|  |  | R: GTCTGGAACCTCAAACCCTATG |  |  |  |  |
|  | *rpl2* | F: TAACGCCTGCCTCTTCACGTTGA | 112 | 60˚C | XM_014137227.1 | - |
|  |  | R: ATGAGGGACCTTGTAGCCAGCAA |  |  |  |  |
| *PCR* | *gh* | F: GGTCCTGAAGCTGCTCCATA | 223 | 59˚C | LOC100136588 | - |
|  |  | R: CCTTGGGGTTTACAGTGCAC |  |  |  |  |
|  | *dsred* | F: CGACATCCCCGACTACAAGA | 153 | 59˚C | KU645306.1 | - |
|  |  | R: TTCACGCCGATGAACTTCAC |  |  |  |  |

Sequences obtained from GenBank (<http://www.ncbi.nlm.nih.gov>)

*fads2d6*, delta-6 fatty acyl desaturase; *fads2d5*, delta-5 fatty acyl desaturase; *elovl2*, fatty acyl elongase 2; *elovl5a*, fatty acyl elongase 5 isoform a; *elovl5b*, fatty acyl elongase isoform b; *fas*, fatty acid synthase; *gpi*, glucose-6-phosphate isomerase; *gys*, glycogen synthase; *acc*, acetyl-CoA carboxylase; *pfk*, phosphofructokinase; *pk*, pyruvate kinase; *g6pd*, glucose-6-phosphate dehydrogenase; *gk5*, glycerol kinase 5; *hprtq*, hypoxanthine phosphoribosyltransferase; *rpl2*; ribosomal protein L2; *gh*, growth hormone; *dsred*, red fluorescent protein.
