# Supplementary material for: An oil containing EPA and DHA from transgenic Camelina sativa to replace marine fish oil in feeds for Atlantic salmon (Salmo salar L.): Effects on intestinal transcriptome, histology, tissue fatty acid profiles and plasma biochemistry
Source: PLoS One. 2017 Apr 12;12(4):e0175415. doi: 10.1371/journal.pone.0175415 (PMC5389825; doi:10.1371/journal.pone.0175415)
Supplement: S3 Table — The presence of glycogen and Goblet cells was assessed using PAS staining, whereas the other parameters were scored with H&E staining. (DOCX) [file pone.0175415.s003.docx]

**Supplementary Table 3**. Description of the semi-quantitative scoring system using different parameters to assess the liver, mid gut and hind gut of Atlantic salmon fed the three experimental feeds containing different lipid sources. The presence of glycogen and Goblet cells was assessed using PAS staining, whereas the other parameters were scored with H&E staining.

| **Tissue** | **Score** | **Parameter** |
| --- | --- | --- |
| Liver |  | **Liver nuclei (LN)** |
|  | 1 | Nuclei tightly granular, small and indistinct |
|  | 2 | Nuclei with abundant dark granules, enlarged nucleoli |
|  | 3 | Nuclei small, dark and pyknotic |
|  |  | **Liver hepatocyte cytoplasm (LHC)** |
|  | 1 | Structured, scattered granules with eosinophilic patches |
|  | 2 | Homogeneous and granular |
|  | 3 | Hyaline, lacking texture, dark and small |
|  |  | **Hepatic vacuolization (HV)** |
|  | 1 | Not observed |
|  | 2 | Few vacuoles |
|  | 3 | Medium presence of vacuoles |
|  | 4 | Severe presence of vacuoles |
|  |  | **Glycogen (G)** |
|  | 1 | Low presence of intracytoplasmatic G |
|  | 2 | Moderate presence |
|  | 3 | High presence |
| Intestine |  | **Mucosa folds length (MF)** |
|  | 1 | Normal height |
|  | 2 | Moderate height |
|  | 3 | Abnormal height |
|  |  | **Goblet cells (GC)** |
|  | 1 | Scattered cells |
|  | 2 | Scattered to moderate numbers and sparsely distributed |
|  | 3 | Moderate numbers and wider distribution of them |
|  | 4 | Increased numbers and more densely distributed |
|  | 5 | High abundant and tightly packed |
|  |  | **Lamina propria (LP)** |
|  | 1 | Normal size LP |
|  | 2 | Normal to moderate size LP |
|  | 3 | Moderate size LP |
|  | 4 | Moderate to increased size LP |
|  | 5 | Large size LP |
|  |  | **Supra-nuclear vacuoles (SNV)** |
|  | 1 | Basal SNV size |
|  | 2 | Moderate increment in SNV size |
|  | 3 | Increase SNV size |
|  | 4 | Onset of SNV extinction |
|  | 5 | No SNV |
|  |  | **Sup-epithelial mucosa (SEM)** |
|  | 1 | Normal SEM |
|  | 2 | Mild increment of SEM |
|  | 3 | Moderate size of SEM |
|  | 4 | Increased size of SEM |
|  | 5 | Large size of SEM |
|  |  | **Eosinophilic granulocytes (EG)** |
|  | 1 | Few EG in SEM |
|  | 2 | Increased number of EG in SEM |
|  | 3 | Increased number of EG |
|  | 4 | Diffused number in LP and SEM |
|  | 5 | Dense EG in SM and LP |
|  |  | **Intra-epithelial lymphocytes (IEL)** |
|  | 1 | Rare IEL |
|  | 2 | Mild presence of IEL |
|  | 3 | Moderate presence of IEL |
|  | 4 | Marked increase in IEL |
|  |  | **Mucosal fold base mitotic activity (MFBMA)** |
|  | 1 | Normal (2-3 mitotic epithelial cells) |
|  | 2 | Moderate (5-10 mitotic cells) |
|  | 3 | High (>10 mitotic cells) |
