# Supplementary material for: An oil containing EPA and DHA from transgenic Camelina sativa to replace marine fish oil in feeds for Atlantic salmon (Salmo salar L.): Effects on intestinal transcriptome, histology, tissue fatty acid profiles and plasma biochemistry
Source: PLoS One. 2017 Apr 12;12(4):e0175415. doi: 10.1371/journal.pone.0175415 (PMC5389825; doi:10.1371/journal.pone.0175415)
Supplement: S4 Table — Features are arranged by functional categories and within them by increasing p value (assessed by Welch t-test). (DOCX) [file pone.0175415.s004.docx]

**Supplementary Table 4**. Annotated transcripts within the 122 features exhibiting common differential expression in midgut of Atlantic salmon fed DCO compared to fish fed either FO or WCO diets. Features are arranged by functional categories and within them by increasing p value (assessed by Welch t-test).

| **KO no** | **FO/DCO** | | **WCO/DCO** | | **Annotation** |
| --- | --- | --- | --- | --- | --- |
|  | p | FC | p | FC |  |
| *Metabolism (26.2 %)* | | |  |  |  |
| *Lipid (15.4 %)* | | |  |  |  |
| K10226 | 0.0026 | 1.55 | 0.0067 | 1.52 | Delta 6 fatty acyl desaturase |
| K10226 | 0.0038 | 1.56 | 0.0056 | 1.55 | Delta 6 fatty acyl desaturase |
| K10226 | 0.0039 | 1.63 | 0.0075 | 1.56 | Delta 6 fatty acyl desaturase |
| K10226 | 0.0047 | 1.57 | 0.0065 | 1.55 | Delta 6 fatty acyl desaturase |
| K10226 | 0.0062 | 1.49 | 0.0106 | 1.43 | Delta 6 fatty acyl desaturase |
| K10226 | 0.0066 | 1.45 | 0.0076 | 1.49 | Delta 6 fatty acyl desaturase |
| K10226 | 0.0085 | 1.48 | 0.0105 | 1.44 | Delta 6 fatty acyl desaturase |
| K08729 | 0.0214 | 1.48 | 0.0411 | 1.44 | Phosphatidylserine synthase 1 |
| K01116 | 0.0243 | 2.56 | 0.0264 | 2.77 | Phosphatidylinositol phospholipase C, gamma-1 |
| K10226 | 0.0272 | 1.63 | 0.0417 | 1.49 | Delta 6 fatty acyl desaturase |
| K00515 | 0.0311 | 1.55 | 0.0289 | 1.50 | Beta-carotene 15,15'-dioxygenase |
| K00515 | 0.0314 | 1.58 | 0.0429 | 1.40 | Beta-carotene 15,15'-dioxygenase |
|  |  |  |  |  |  |
| *Amino acid (4.6 %)* | | |  |  |  |
| K00757 | 0.0021 | -1.61 | 0.0065 | -1.37 | Uridine phosphorylase |
| K03021 | 0.0069 | 1.55 | 0.0121 | 1.58 | DNA-directed RNA polymerase III subunit RPC2 |
| K00757 | 0.0262 | 2.06 | 0.0288 | 1.81 | Uridine phosphorylase |
|  |  |  |  |  |  |
| *Energy metabolism (1.5 %)* | | | |  |  |
| K02261 | 0.0100 | 1.76 | 0.0439 | 1.61 | Cytochrome c oxidase subunit 2 |
|  |  |  |  |  |  |
| *Other metabolic process (1.5 %)* | | | |  |  |
| K00485 | 0.0097 | 1.75 | 0.0284 | 1.64 | Dimethylaniline monooxygenase (N-oxide forming) |
|  |  |  |  |  |  |
| *Translation (3.1 %)* | | | |  |  |
| K14572 | 0.0160 | 2.09 | 0.0124 | 2.20 | Midasin |
| K03260 | 0.0377 | -1.81 | 0.0341 | -2.17 | Translation initiation factor 4G |
|  |  |  |  |  |  |
| *Transport and catabolism (12.3 %)* | | | |  |  |
| K12485 | 0.0166 | 1.94 | 0.0417 | 1.58 | Rab11 family-interacting protein 3/4 |
| K14683 | 0.0228 | 1.78 | 0.0249 | 1.64 | Solute carrier family 34 |
| K14714 | 0.0452 | 1.44 | 0.0349 | 1.42 | Solute carrier family 39 |
| K14683 | 0.0096 | 1.60 | 0.0188 | 1.41 | Solute carrier family 34 |
| K14683 | 0.0171 | 1.58 | 0.0159 | 1.48 | Solute carrier family 34 |
| K14702 | 0.0329 | 1.51 | 0.0297 | 1.37 | Solute carrier family 26 |
| K12479 | 0.0303 | -1.45 | 0.0189 | -1.52 | Vacuolar protein sorting-associated protein 45 |
| K10413 | 0.0014 | -1.95 | 0.0232 | -1.66 | Dynein heavy chain 1, cytosolic |
|  |  |  |  |  |  |
| *Signalling (6.2 %)* | | | | |  |
| K13024 | 0.0354 | -2.06 | 0.0233 | -2.83 | Inositol-hexakisphosphate/diphosphoinositol-pentakisphosphate 1-kinase |
| K03068 | 0.0052 | 2.03 | 0.0397 | 1.84 | Low density lipoprotein receptor-related protein 5/6 |
| K19614 | 0.0024 | 1.62 | 0.0104 | 1.68 | Pigment epithelium-derived factor |
| K04469 | 0.0243 | 1.41 | 0.0160 | 1.50 | Nuclear factor of kappa light polypeptide gene enhancer |
|  |  |  |  |  |  |
| *Protein folding (4.6 %)* | | | |  |  |
| K09540 | 0.0019 | -1.78 | 0.0144 | -1.53 | Translocation protein SEC63 |
| K10615 | 0.0117 | 1.58 | 0.0386 | 1.39 | E3 ubiquitin-protein ligase HERC4 |
| K10576 | 0.0118 | 1.44 | 0.0030 | 1.55 | Ubiquitin-conjugating enzyme E2 H |
|  |  |  |  |  |  |
| *Transcription (3.1. %)* | | | |  |  |
| K12893 | 0.0055 | -1.80 | 0.0348 | -1.51 | Splicing factor, arginine/serine-rich 4/5/6 |
| K09040 | 0.0224 | 1.59 | 0.0021 | 1.88 | Nuclear factor erythroid 2-related factor 1/3 |
|  |  |  |  |  |  |
| *Digestive system (10.8 %)* | | | |  |  |
| K14710 | 0.0009 | 2.58 | 0.0417 | 1.56 | Solute carrier protein 30 |
| K01395 | 0.0120 | 1.83 | 0.0335 | 1.89 | Meprin A |
| K19721 | 0.0292 | 1.82 | 0.0203 | 2.12 | Collagen, type V/XI/XXIV/XXVII, alpha |
| K05641 | 0.0289 | 1.52 | 0.0045 | 1.94 | ATP-binding cassette, subfamily A (ABC1), member 1 |
| K00678 | 0.0096 | 1.50 | 0.0456 | 1.56 | Phosphatidylcholine-retinol O-acyltransferase |
| K08760 | 0.0315 | 1.40 | 0.0171 | 1.41 | Apolipoprotein A-IV |
| K14463 | 0.0216 | 1.37 | 0.0333 | 1.31 | microsomal triglyceride transfer protein large subunit |
|  |  |  |  |  |  |
| *Immune system (12.3 %)* | | | |  |  |
| K15412 | 0.0068 | -1.62 | 0.0381 | -1.52 | Complement factor properdin |
| K01369 | 0.0497 | -2.78 | 0.0418 | -1.84 | Legumain |
| K01320 | 0.0001 | 2.31 | 0.0089 | 1.78 | Coagulation factor VII |
| K03999 | 0.0253 | -1.91 | 0.0130 | -2.47 | Complement component 8 subunit gamma |
| K01365 | 0.0034 | 1.61 | 0.0085 | 1.48 | Cathepsin L |
| K12649 | 0.0201 | 1.48 | 0.0487 | 1.36 | ATP-dependent RNA helicase DHX58 |
| K06261 | 0.0302 | 1.43 | 0.0374 | 1.42 | Platelet glycoprotein Ib alpha chain |
| K05464 | 0.0364 | 1.52 | 0.0114 | 1.78 | Growth arrest-specific 6 |
|  |  |  |  |  |  |
| *Miscellaneous or unknown function (21.5 %)* | | | | |  |
| K05729 | 0.0036 | 14.42 | 0.0367 | 8.11 | Rho guanine nucleotide exchange factor 6 |
| K16493 | 0.0008 | 3.01 | 0.0159 | 2.29 | Protocadherin alpha |
| K07739 | 0.0407 | -2.66 | 0.0106 | -3.72 | Elongator complex protein 3 |
| K17934 | 0.0013 | -2.39 | 0.0117 | -2.02 | NAPDH oxidase organizer 1 |
| K17046 | 0.0435 | 1.87 | 0.0268 | 1.81 | Protein DEK |
| K18264 | 0.0230 | 1.84 | 0.0164 | 1.86 | Integral membrane protein 2B |
| K11982 | 0.0128 | -1.82 | 0.0496 | -1.62 | E3 Ubiquitin-protein ligase |
| K18595 | 0.0006 | -1.80 | 0.0363 | -1.57 | Echinoderm microtubule-associated protein-like 1/2 |
| K10647 | 0.0056 | 1.75 | 0.0318 | 1.59 | Midline 2 |
| K04309 | 0.0200 | 1.56 | 0.0432 | 1.45 | Leucine-rich repeat-containing G preotin-coupled receptor 4 |
| K04917 | 0.0159 | -1.53 | 0.0381 | -1.40 | Potassium channel subfamily K member 6 |
| K16727 | 0.0421 | 1.39 | 0.0406 | 1.46 | Rho guanine nucleotide exchange factor 10 |
| K19728 | 0.0201 | 1.38 | 0.0174 | 1.49 | Familial hemophagocytic lymphohistiocytosis (FHPL) |
| K20067 | 0.0050 | 1.38 | 0.0064 | 1.37 | Stonin-1/2 |
