# Supplementary material for: An oil containing EPA and DHA from transgenic Camelina sativa to replace marine fish oil in feeds for Atlantic salmon (Salmo salar L.): Effects on intestinal transcriptome, histology, tissue fatty acid profiles and plasma biochemistry
Source: PLoS One. 2017 Apr 12;12(4):e0175415. doi: 10.1371/journal.pone.0175415 (PMC5389825; doi:10.1371/journal.pone.0175415)
Supplement: S5 Table — Features are arranged by functional categories and within them by increasing p value (assessed by Welch t-test). (DOCX) [file pone.0175415.s005.docx]

**Supplementary Table 5**. Annotated transcripts in the 92 features exhibiting common differential expression in hind gut of Atlantic salmon fed DCO compared to fish fed either FO or WCO diets. Features are arranged by functional categories and within them by increasing p value (assessed by Welch t-test).

| **KO no** | **FO/DCO** | | **WCO/DCO** | | **Annotation** |
| --- | --- | --- | --- | --- | --- |
|  | p | FC | p | FC |  |
| *Metabolism (10.3 %)* | | |  |  |  |
| K03844 | 0.0396 | -1.47 | 0.0239 | -1.49 | Alpha-1,2-mannosyltransferase |
| K11158 | 0.0004 | +2.33 | 0.0365 | +1.65 | Retinoid isomerohydrolase |
| K00485 | 0.0471 | +2.61 | 0.0419 | +3.12 | Dimethylaniline monooxygenase (N-oxide forming) |
|  |  |  |  |  |  |
| *Transcription (13.8 %)* | | | |  |  |
| K12893 | 0.0014 | 1.46 | 0.0354 | +1.34 | Splicing factor, arginine/serine-rich 4/5/6 |
| K12811 | 0.0101 | 2.07 | 0.0453 | +1.79 | ATP-dependent RNA helicase DDX46/PRP5 |
| K12843 | 0.0143 | 2.10 | 0.0173 | +2.21 | U4/U6 small nuclear ribonucleoprotein PRP3 |
| K12893 | 0.0373 | 1.67 | 0.0392 | +1.55 | Splicing factor, arginine/serine-rich 4/5/6 |
|  |  |  |  |  |  |
| *Translation (17.2 %)* | | | |  |  |
| K14305 | 0.0072 | +1.30 | 0.0320 | +1.79 | Nuclear pore complex protein Nup43 |
| K14299 | 0.0245 | +1.51 | 0.0071 | +1.60 | Nucleoporin SEH1 |
| K01867 | 0.0282 | +1.53 | 0.0438 | +1.52 | Tryptophanyl-tRNA synthetase |
| K02997 | 0.0341 | -1.83 | 0.0235 | -1.87 | Small subunit ribosomal protein S9e |
| K02978 | 0.0434 | +1.70 | 0.0452 | +1.50 | Small subunit ribosomal protein S27e |
|  |  |  |  |  |  |
| *Protein folding (3.4 %)* | | | | |  |
| K10609 | 0.0359 | +1.50 | 0.0414 | +1.49 | Cullin 4 |
|  |  |  |  |  |  |
| *Signalling (34.5 %)* | | | |  |  |
| K08341 | 0.0092 | -1.43 | 0.0013 | -1.39 | GABA(A) receptor-associated protein |
| K04508 | 0.0149 | +3.31 | 0.0154 | +2.47 | Transducin (beta)-like 1 |
| K04683 | 0.0151 | +2.40 | 0.0346 | +2.79 | Transcription factor Dp-1 |
| K04688 | 0.0170 | +1.53 | 0.0287 | +1.36 | p70 ribosomal S6 kinase |
| K06793 | 0.0174 | +1.64 | 0.0286 | +1.48 | Versican core protein |
| K06092 | 0.0254 | +2.80 | 0.0341 | +2.79 | InaD-like protein |
| K04630 | 0.0364 | -1.42 | 0.0395 | -1.62 | Guanine nucleotide-binding protein G(i) subunit alpha |
| K06585 | 0.0431 | -1.46 | 0.0262 | -1.58 | Integrin alpha 9 |
| K13302 | 0.0444 | -1.80 | 0.0075 | -2.36 | Serum/glucocorticoid-regulated kinase 1 |
| K05637 | 0.0452 | +1.71 | 0.0130 | +1.97 | Laminin, alpha 1/2 |
|  |  |  |  |  |  |
| *Immune system (3.4 %)* | | | |  |  |
| K12804 | 0.0218 | +1.65 | 0.0312 | +1.43 | Proline-serine-threonine phosphatase interacting protein 1 |
|  |  |  |  |  |  |
| *Endocrine system (3.4 %)* | | | | |  |
| K08794 | 0.0267 | -4.44 | 0.0079 | -3.81 | Calcium/calmodulin-dependent protein kinase I |
|  |  |  |  |  |  |
| *Miscellaneous or unknown function (13.8 %)* | | | | |  |
| K05764 | 0.0091 | -1.36 | 0.0211 | -1.79 | Thymosin, beta 4 |
| K06675 | 0.0297 | -1.45 | 0.0172 | -1.51 | Structural maintenance of chromosome 4 |
| K05705 | 0.0303 | +1.39 | 0.0096 | +1.41 | Tyrosine-protein kinase Yes |
| K06618 | 0.0409 | +1.38 | 0.0488 | +1.32 | Retinoblastoma-associated protein |
