# Supplementary material for: An oil containing EPA and DHA from transgenic Camelina sativa to replace marine fish oil in feeds for Atlantic salmon (Salmo salar L.): Effects on intestinal transcriptome, histology, tissue fatty acid profiles and plasma biochemistry
Source: PLoS One. 2017 Apr 12;12(4):e0175415. doi: 10.1371/journal.pone.0175415 (PMC5389825; doi:10.1371/journal.pone.0175415)
Supplement: S6 Table — (DOCX) [file pone.0175415.s006.docx]

**Supplementary Table 6**. Validation of microarray results by qPCR

|  | DCO vs WCO | | | |
| --- | --- | --- | --- | --- |
|  | Microarray | | qPCR | |
|  | p | FC | p | FC |
| *fads2d6* | 0.020 | -1.49 | 0.002 | -1.89 |
| *fads2d5* | 0.019 | -1.47 | 0.042 | -1.67 |
| *elovl2* | >0.05 | -1.45 | >0.05 | -1.54 |
| *elovl5a* | >0.05 | -1.41 | >0.05 | -1.32 |
| *elovl5b* | 0.035 | -2.40 | >0.05 | -1.39 |
| *g6pdh* | 0.03 | +1.30 | 0.028 | +1.40 |

Data shown are the fold-changes (FC) and p values (assessed by REST 2009) between expression levels in midgut of salmon fed DCO and WCO diets in comparison with microarray data. *fads2d6*, delta-6 fatty acyl desaturase; *fads2d5*, delta-5 fatty acyl desaturase; *elovl2*, fatty acyl elongase 2; *elovl5a*, fatty acyl elongase 5 isoform a; *elovl5b*, fatty acyl elongase isoform b; *g6pdh*, glucose-6-phosphate dehydrogenase.
