# Supplementary material for: An oil containing EPA and DHA from transgenic Camelina sativa to replace marine fish oil in feeds for Atlantic salmon (Salmo salar L.): Effects on intestinal transcriptome, histology, tissue fatty acid profiles and plasma biochemistry
Source: PLoS One. 2017 Apr 12;12(4):e0175415. doi: 10.1371/journal.pone.0175415 (PMC5389825; doi:10.1371/journal.pone.0175415)
Supplement: S1 Fig — n-3 LC-PUFA, omega-3 long chain polyunsaturated fatty acids (sum of 20:4n-3, 20:5n-3, 22:5n-3 and 22:6n-3). (DOCX) [file pone.0175415.s007.docx]

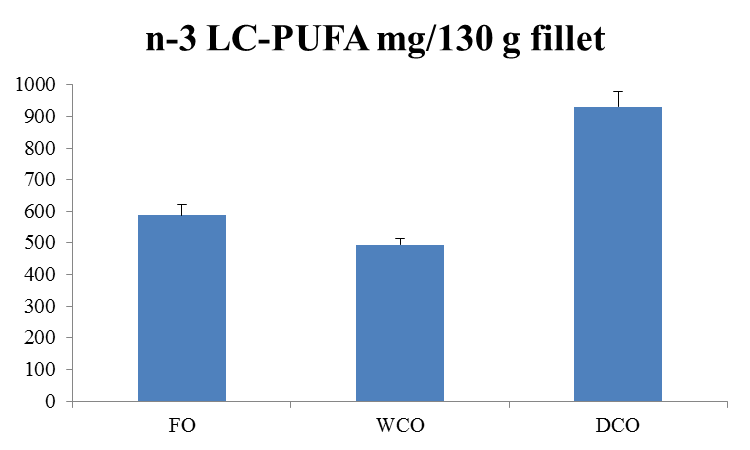


**Supplementary Figure 1**. Absolute n-3 LC-PUFA contents (mg) per portion of fillet (130 g). n-3 LC-PUFA, omega-3 long chain polyunsaturated fatty acids (sum of 20:4n-3, 20:5n-3, 22:5n-3 and 22:6n-3).
